# Supplementary material for: Dissociation of nanosilicates induces downstream endochondral differentiation gene expression program
Source: Sci Adv. 2022 Apr 27;8(17):eabl9404. doi: 10.1126/sciadv.abl9404 (PMC9045714; doi:10.1126/sciadv.abl9404)
Supplement: 20220427-1 [file sciadv.abl9404.v1.pdf]

HEALTH AND MEDICINE

Dissociation of nanosilicates induces downstream endochondral differentiation gene expression program

Copyright © 2022 The Authors, some rights reserved; exclusive licensee American Association for the Advancement of Science. No claim to original U.S. Government Works. Distributed under a Creative Commons Attribution License 4.0 (CC BY).

Anna M. Brokesh[1]†, Lauren M. Cross[1]†, Anna L. Kersey[1], Aparna Murali[1], Christopher Richter[1], Carl A. Gregory[1,2], Irtisha Singh[1,2,3]*, Akhilesh K. Gaharwar[1,3,4,5]*

Bioactive materials harness the body's innate regenerative potential by directing endogenous progenitor cells to facilitate tissue repair. Dissolution products of inorganic biomaterials provide unique biomolecular signaling for tissue-specific differentiation. Inorganic ions (minerals) are vital to biological processes and play crucial roles in regulating gene expression patterns and directing cellular fate. However, mechanisms by which ionic dissolution products affect cellular differentiation are not well characterized. We demonstrate the role of the inorganic biomaterial synthetic two-dimensional nanosilicates and its ionic dissolution products on human mesenchymal stem cell differentiation. We use whole-transcriptome sequencing (RNA-sequencing) to characterize the contribution of nanosilicates and its ionic dissolution products on endochondral differentiation. Our study highlights the modulatory role of ions in stem cell transcriptome dynamics by regulating lineage-specific gene expression patterns. This work paves the way for leveraging biochemical characteristics of inorganic biomaterials to direct cellular processes and promote in situ tissue regeneration.

INTRODUCTION

In situ tissue regeneration approaches use bioactive materials to recruit endogenous stem cells to injury sites and promote new tissue formation (*1*). Recent strategies focus on the development of bioactive materials to elicit the innate regenerative processes of the human body. This approach helps to direct the fate and behavior of endogenous cells and subsequent tissue healing and regeneration. Bioactive materials can exert proregenerative characteristics by their unique physical and chemical attributes. Specifically, the use of physical and structural characteristics such as mechanical cues, topography, surface roughness, hydrophobic characteristics, and surface charge have been leveraged to modulate cellular processes (*1*). More recently, biomaterial design advancements leverage biochemical attributes, capitalizing on degradation products to direct tissue healing and regeneration. This approach can direct cellular differentiation and promote tissue regeneration without the use of growth factors or other small-molecule drugs.

Inorganic biomaterials can promote tissue regeneration via the release of inorganic ions (minerals) (*2*–*4*). Several inorganic ions have previously been implicated in directing cellular processes (*5*–*12*). For example, zinc ions direct neural processes (*10*, *11*), vascularization (*13*), and osteogenesis (*12*). Gold ions and gold nanoparticles may activate processes key to osteogenesis (*8*). Copper ions have been suggested to promote vascularization (*9*). Silicon (Si) contributes to processes key to cartilage and connective tissue (*7*) and may also play a role in bone health because of its involvement in collagen synthesis (*14*). Magnesium (Mg) also supports chondrogenesis (*6*) and osteogenesis (*15*) in human mesenchymal stem cells (hMSCs), although the mechanism for this action is not well understood. Lithium (Li) plays a role in both chondrogenesis and osteogenesis by distinctly affecting Wingless (Wnt)/β-catenin signaling (*16*–*18*). In addition, in well-known biomaterials, such as bioglass, hydroxyapatite, and β-tricalcium phosphate, the release of calcium (Ca) and phosphate (PO_4^{3-}) ion dissolution products is implicated in the induction of osteogenesis. Although ionic dissolution products of biomaterials are suggested to control widespread cellular processes, the exact contribution of individual ions and the underlying mechanisms to direct stem cell differentiation and tissue healing have not been explored. Further research into the effects of ionic dissolution products released from bioactive materials can reveal avenues to tailor such bioactive materials toward specific tissue regeneration applications.

An emergent class of mineral-based nanomaterials, synthetic nanosilicates (nSi) $\{Na^{+}_{0.7}[(Mg_{5.5}Li_{0.3})Si_8O_{20}(OH)_4]^{-0.7}$; Laponite XLG$\}$, has been shown to induce stem cell differentiation in the absence of growth factors (*19*–*23*). These are disk-shaped nanoparticles with 20- to 50-nm diameter and 1- to 2-nm thickness. Nanosilicates demonstrate high cytocompatibility with hMSCs and show innate bioactivity (*23*, *24*). The release of ionic dissolution products from nSi may contribute to the bioactive nature of this and other inorganic biomaterials. While these studies have produced encouraging results for nSi innate bioactivity, the underlying molecular mechanisms of inducing stem cell differentiation remains elusive.

Here, we attempt to decipher the molecular mechanisms of nSi bioactivity and the role of ionic dissolution products on hMSC differentiation. Our central hypothesis is that synthetic nSi dissociate into their individual components (Si, Mg, and Li) under physiological conditions and activate unique signaling pathways, which, in turn, induce differentiation. We determined the release profile of ionic dissolution products from nSi and characterize their ability to promote osteogenesis in hMSCs. Furthermore, we leveraged the computational power of whole-transcriptomic sequencing [RNA sequencing (RNA-seq)] to evaluate the effect of nSi and ionic dissolution products on the transcriptome dynamics of hMSCs. RNA-seq provides an unbiased overview of the gene expression profile and

[1]Department of Biomedical Engineering, Dwight Look College of Engineering, Texas A&M University, College Station, TX 77843, USA. [2]Department of Molecular & Cellular Medicine, Texas A&M University Health Science Center, Bryan, TX 77807-3260, USA. [3]Interdisciplinary Program in Genetics, Texas A&M University, College Station, TX 77843, USA. [4]Center for Remote Health Technologies and Systems, Texas A&M University, College Station, TX 77843, USA. [5]Department of Material Science and Engineering, Dwight Look College of Engineering, Texas A&M University, College Station, TX 77843, USA.
*Corresponding author. Email: gaharwar@tamu.edu (A.K.G.); isingh@tamu.edu (I.S.)
†These authors contributed equally to this work.

overcomes the limitations of conventional approaches such as the subjectively used polymerase chain reaction or microarrays. Furthermore, the holistic nature of RNA-seq can be used to uncover biological processes and transcriptome expression that would otherwise go unnoticed by traditional approaches. This high-throughput readout of the molecular state of mesenchymal stem cells provides crucial insight into the late-stage (21-day) nuances of nSi-induced osteogenesis. We used this information to learn about the role of ions in directing cellular processes and lineage commitment. Our approach delineates the contribution of nSi and its ionic dissolution products in mesenchymal stem cell differentiation.

RESULTS AND DISCUSSION

Ionic dissolution of nSi in physiological conditions

Synthetic nSi $\{Na^{+}_{0.7}[(Mg_{5.5}Li_{0.3})Si_8O_{20}(OH)_4]^{-0.7}\}$ are composed of layered structures of octahedral Mg and Li ions sandwiched between tetrahedral Si ions (Fig. 1A). The isoelectric point of nSi is pH ~10 (*25*, *26*); thus, we hypothesize that at lower pH (<9), nSi dissociate into ionic dissolution products [Li^+, $Si(OH)_4$, and Mg^{2+}]. As nSi are internalized by hMSCs predominantly via clathrin-mediated endocytosis (*19*), the dissociation of nSi likely occurs in the endosome because of the low pH 5.5. In addition, the pH of the extracellular environment is pH ~7.4, which could also facilitate nSi dissociation. Therefore, we investigated nSi dissociation at three different pH values (~5.5, ~7.4, and ~10) and determined the concentration of the dissociation products [Li^+, $Si(OH)_4$, and Mg^{2+}] through inductively coupled plasma mass spectrometry (ICP-MS).

Specifically, ion dissociation from a nSi solution [50 μg/ml dispersed in 10 ml of deionized (DI) water] was monitored for 21 days at pH 5.5 (mimicking endosome microenvironment), pH 7.4 (mimicking extracellular microenvironment), and pH 10 (point of zero charge of nSi) (*25*). As expected, at the lower pH (5.5 and 7.4), higher concentrations of ions were present, supporting our hypothesis that nSi dissociate at physiological pH (Fig. 1B). The concentration of Li was the lowest (0.09 ± 0.04 μg/ml) compared with Mg (2.19 ± 0.99 μg/ml) and Si (5.89 ± 2.65 μg/ml) over 21 days at pH 5.5. While the concentration of Li released was the lowest of the three ions across all pH, the percent released of Li (63.94% at pH 5.5) was the greatest. Li ions release more rapidly than Mg or Si ions, as Li ions are bound by a

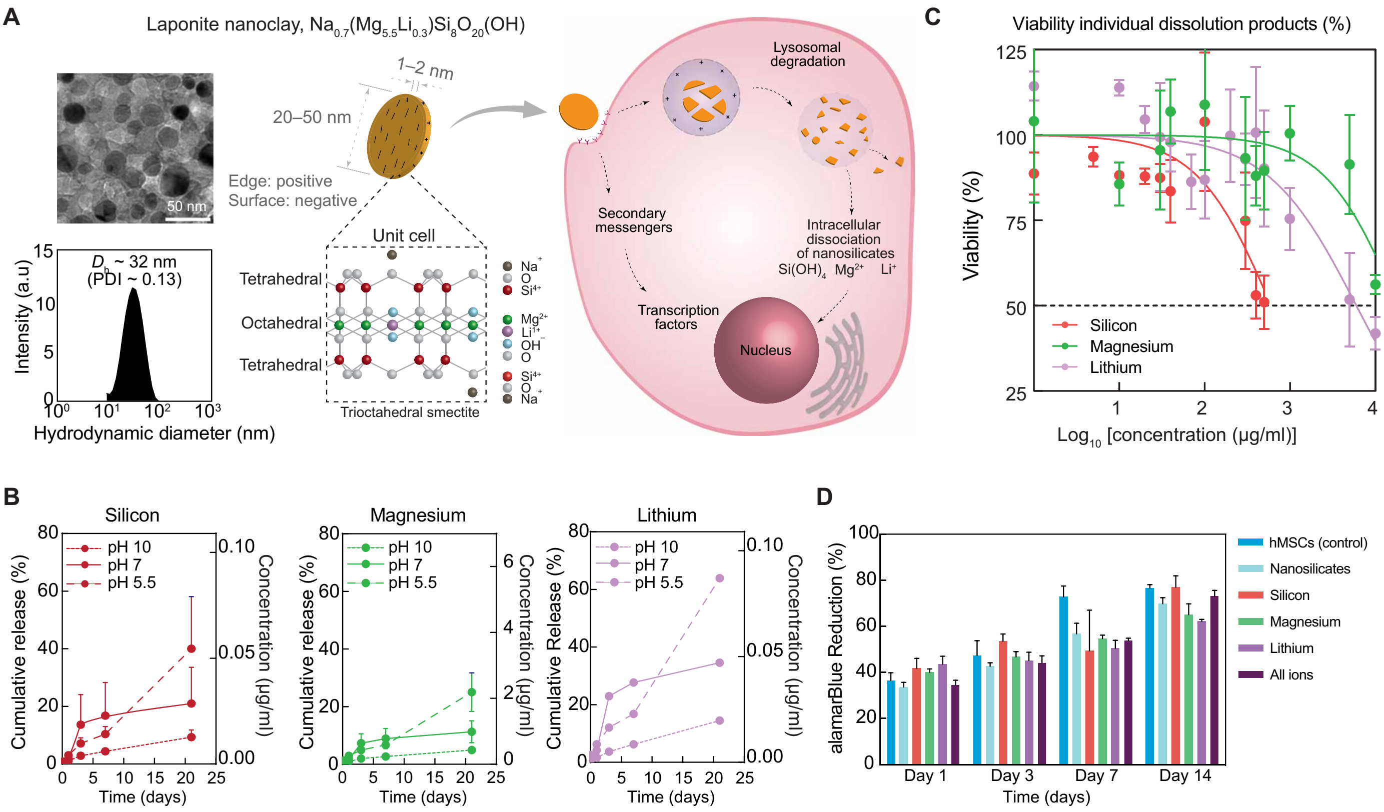

Fig. 1. Structure, physiological stability, and cellular compatibility of nanosilicate. (**A**) Nanosilicates (nSi) are plate-like poly-ions composed of simple or complex salts of silicic acids with a heterogeneous charge distribution and patchy interactions. Transmission electron microscopy (TEM) images show the size of these nanosilicates to be between 20 and 50 nm in diameter. Dynamic light scattering (DLS) shows the hydrodynamic diameter (D_h) of nanosilicates is ~32 nm in aqueous conditions with polydispersity index (PDI) ~0.13. The schematic shows the potential interactions of nanosilicates with cells. nSi dissociate into individual ions once introduced to a physiological microenvironment (pH <9). a.u., arbitrary units. (**B**) Dissolution of nanosilicates was monitored using inductively coupled plasma mass spectrometry (ICP-MS) in different pH to mimic the extracellular (pH ~7.4) and intracellular microenvironments (pH ~5.5). nSi are expected to be stable at pH ~10 and thus used as control. (**C**) The effect of nanosilicates and its ionic dissolution products (silicon, magnesium, and lithium) on cellular viability was evaluated using MTT assay. Three technical replicates were used for each condition. Half-maximal inhibitory concentration (IC_{50}) is labeled at 50% viability. Concentrations of released ions from nSi fall well below the IC_{50} value. (**D**) Long-term cellular viability after treatment with nanoparticles and its ionic dissolution products was assessed using alamarBlue assay to detect metabolically active cells ($n = 3$).

hydroxide (OH^-) rather than an oxide. Previous studies have reported that, after nSi are dispersed in water, OH^- ions dissociate from the edge (*25*, *26*). Specifically, at lower pH, nSi attempt to restabilize a basic pH via release of OH^-. In addition, monovalent Li is less stable than divalent Mg or tetravalent Si, so release of Li occurs more rapidly. Si ion release (~40% release at pH 5.5) was also observed to be greater than that of magnesium (~25% release at pH 5.5), as Si ions are present on the outer layer and are more susceptible to dissociation. After 21 days, the average percent release of individual ions at physiologically relevant pH (7.4 and 5.5) were ~31% (4.49 ± 1.98 μg/ml) Si, ~49% (0.067 ± 0.03 μg/ml) Li, and ~18% (1.59 ± 0.85 μg/ml) Mg. To further investigate the effect of these individual ions on cell behavior, the average percent release of the ions at physiological pH after 21days was chosen for subsequent studies.

Previous studies have demonstrated high cytocompatibility [half-maximal inhibitory concentration (IC_{50}), ~1 to 4 mg/ml] of nSi (*19*, *24*). As nSi dissociate into individual ions, we investigated the effect of individual ionic products (Li, Si, and Mg) on cell viability and proliferation. Li and Mg ensured 80% cell viability over a wide concentration range, up to 1 mg/ml, while Si only allowed for 80% cell viability at concentrations ~100 μg/ml (Fig. 1C). These concentrations were nearly 100 to 1000 times greater than those observed because of nSi dissociation (4.49 μg/ml Si, 0.067 μg/ml Li, and 1.59 μg/ml Mg) (Fig. 1B). To investigate the effect of the individual ions on cell health, hMSCs were treated with ions that were released after 21 days (~4.49 μg/ml of Si, 0.067 μg/ml of Li and 1.59 μg/ml of Mg). No significant difference in viability was observed compared with the untreated control supporting nSi, and individual ions did not negatively affect hMSC health (Fig. 1D). These results support that nSi dissociation products are cytocompatible and facilitate normal cellular metabolism and proliferation over time.

Ionic dissolution products of nSi induce endochondral differentiation

Bone tissue morphogenesis can be crudely categorized into two groups: intramembranous ossification and endochondral ossification (*27*). Intramembranous ossification is considered the direct osteogenic differentiation of endogenous stem cells to osteoblast cells, which deposit mineralized bone matrix and promote bone tissue regeneration (*28*). Use of dexamethasone or bone morphogenic protein 2 (BMP2) for in situ bone regeneration results in intramembranous ossification. Endochondral ossification first relies on the production of a hyaline cartilaginous matrix, which is then replaced by bone tissue through several mechanisms (*29*), of which the proteinases and collagenases responsibly originate from several cell types. The latter process is typical of long bone fracture repair, whereas the former is akin to repair in cranial bones and small bone deficits with a low degree of micromotion (*28*). Recent reports suggest that stem cells in this environment differentiate into chondrocytes, become hypertrophic, and at this point transdifferentiate into osteoblasts to facilitate the deposition of mineralized matrix (*27*).

While this process is still being characterized, there are certain indicators of this process. First, presence of the transcription factor SRY-box transcription factor 9 (*SOX9*) at early time points is observed (*30*), in hMSCs around day 7 of differentiation (*31*), which controls the production of cartilaginous extracellular matrix (ECM) consisting of collagen II, glycoproteins such as glycosaminoglycans (GAGs), and, more specifically, markers such as cartilage oligomeric matrix protein (COMP) and aggrecan (ACAN). This transcription factor can be activated through a few different mechanisms: fibroblast growth factor 2 (FGF2), transforming growth factor–β1 (TGF-β1), Wnt, sonic hedgehog (Shh), Indian hedgehog (Ihh), and hypoxia [hypoxia-inducible factor (HIF)] pathways (*30*). Furthermore, during later stages, chondrocytes undergo hypertrophy and express collagen X (*27*, *32*), which is a marker previously found to coincide with the onset of ossification (*33*, *34*). Investigations have suggested that these hypertrophic chondrocytes transdifferentiate into osteoblast-like cells, as they begin to express markers for mineralization such as bone sialoprotein (BSP), osteocalcin (OCN), and collagen I (*35*). This process has not been fully characterized, and many signaling molecules have been suggested to play a role in this transition. However, the activation of runt-related transcription factor 2 (*RUNX2*), the well-known osteospecific transcription factor, among other osteospecific glycoproteins has been identified at late stages during this transitionary period from hypertrophic chondrocytes to preosteoblasts (*36*, *37*).

Earlier studies highlight that nSi have the ability to direct the differentiation of hMSCs toward osteogenic and chondrogenic lineages (*19*). This observation led us to hypothesize that nSi can cause endochondral differentiation in hMSCs, which go through a transitionary period from chondroblasts to osteoblasts at day 21 (Fig. 2A). In addition, our results show that nSi dissociate into ionic dissolution products at physiological conditions, both intracellularly and in the extracellular microenvironment. On the basis of previous results and our observation, it is expected that ionic dissolution products of nSi might be responsible for endochondral ossification. To test this hypothesis, we treated seeded hMSCs with different conditions (nSi, Li, Si, Mg, and all ions) over 21 days and evaluated key markers for endochondral differentiation. Ion concentrations were selected from ICP-MS data, in which an average of ~31% (4.49 ± 1.98 μg/ml) Si, ~49% (0.067 ± 0.03 μg/ml) Li, and ~18% (1.59 ± 0.85 μg/ml) Mg were released after 21 days. Individual ions were replenished every 3 to 4 days with media changes. After 7 and 14 days, alkaline phosphatase (ALP)—an early marker for hMSC differentiation—production was evaluated via surface staining and an ALP p-nitrophenyl phosphate (PNPP) assay (Fig. 2B and fig. S1). After 7 days, treatment with nSi, Si, and the combination of ions resulted in nearly a twofold increase in ALP production compared with the untreated control (normal growth media supplemented with 10 mM β-glycerophosphate and 50 μM ascorbic acid) (***$P < 0.001$). After 14 days, treatment with Si resulted in similar ALP protein production to the nSi. In addition, after 14 days of treatment, individual ions along with the combination of ions resulted in significantly greater ALP activity compared with the untreated control (***$P < 0.001$).

To evaluate the effect of individual ion on endochondral specific proteins, we performed Western blot on day 14. Specifically, ALP, OCN, osteopontin (OPN), collagen type I (COL1A1), ACAN, type 10 collagen (COL10A1), and COMP expression were evaluated (Fig. 2C). Nanosilicate treatment resulted in a nearly twofold increase in protein expression of OPN (*$P < 0.05$) compared with the untreated control. Similarly, the addition of Si resulted in a nearly threefold higher OPN production (**$P < 0.01$) and twofold higher ALP production compared with the untreated control. While Mg increased OCN production nearly twofold, no significant increase in ALP and OPN was observed in comparison with the control. Treatment with nSi resulted in a nearly 15-fold increase in COL1A1 production (*$P < 0.05$) as evidenced by the presence of protein bands and quantification compared with the untreated control. A significant

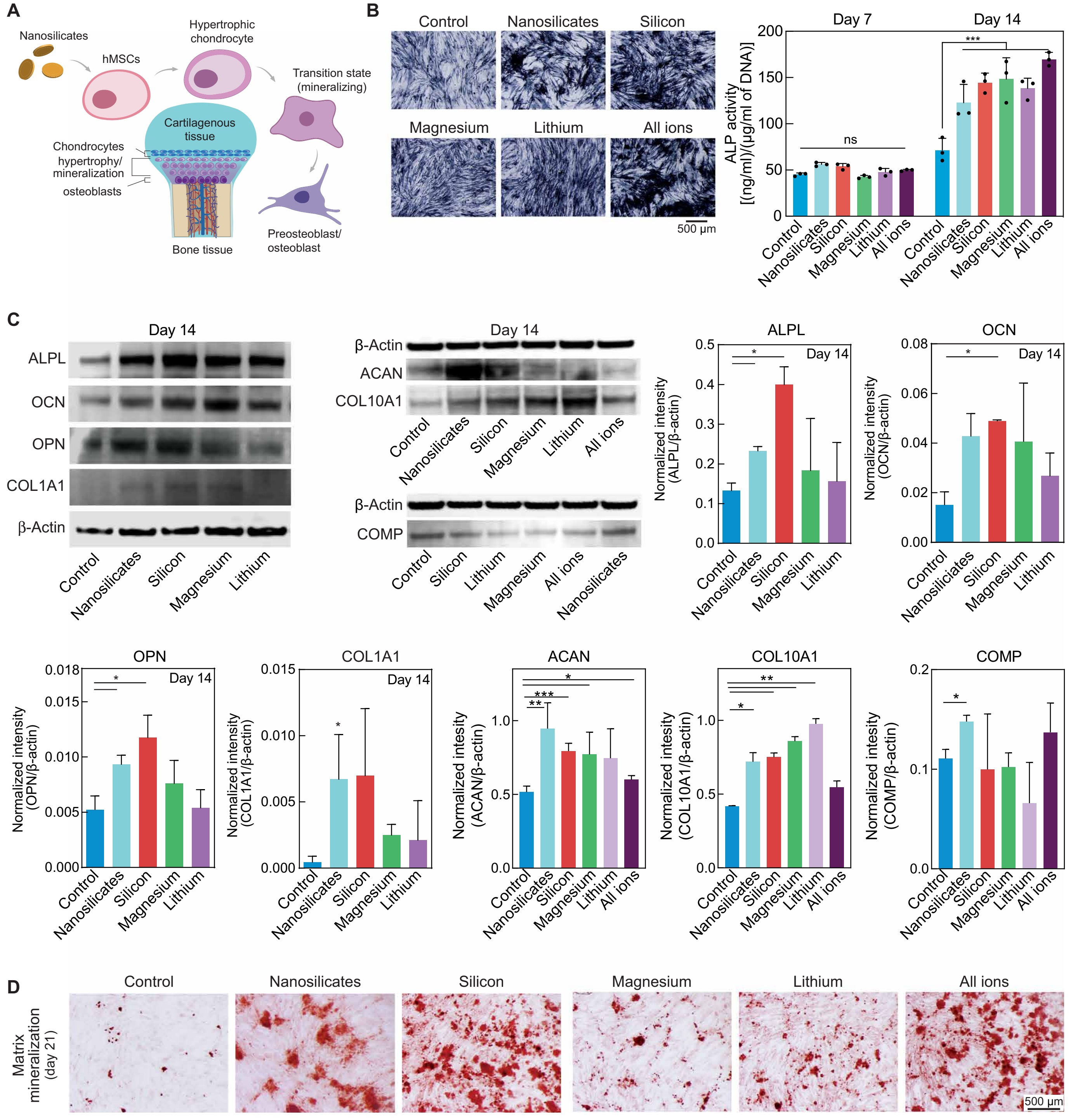

Fig. 2. Effect of nanosilicates and its ionic dissolution products on osteogenic differentiation of hMSCs. (**A**) Schematic depicting nanosilicate-induced endochondral differentiation of hMSCs by first causing chondrogenic differentiation and then a transition to preosteoblastic phenotype. (**B**) Qualitative assessment of production of alkaline phosphatase (ALP) (day 14) due to treatment with nanosilicates and its ionic dissolution products. Blue precipitates represent intracellular ALP (stained using p-nitrophenyl phosphate, disodium salt). hMSCs cultured in osteoconductive media is used as controls. ALP activity is evaluated using kinetic assay for days 7 and 14. Statistical comparison between samples is performed using one-way ANOVA and Dunnett's multiple comparison test, with a single pooled variance ($n = 3$; ns, not significant; $^{***}P < 0.001$). (**C**) Effect of nanosilicate and its ionic dissolution products on production of endochondral-specific proteins—alkaline phosphatase (ALP), osteocalcin (OCN), osteopontin (OPN), collagen type I alpha 1 (COL1A1), aggrecan (ACAN), type 10 collagen (COL10A1), and cartilage oligomatrix protein (COMP) are determined using Western blot (day 14). Housekeeping protein (β-actin) is used as internal control. The amount of proteins was normalization by housekeeping protein (β-actin) for quantitative assessment ($^{*}P < 0.05$; $^{**}P < 0.01$; $^{***}P < 0.001$). (**D**) The effect of nanosilicates and its ionic dissolution products was evaluated on production of mineralized extracellular matrix (ECM) on day 21. Red precipitates represent calcium deposits (stained using Alizarin Red S).

increase in ACAN in all samples was observed compared with the negative control (hMSCs only). nSi significantly (*$P < 0.05$) increased the expression of COMP compared with the negative control at day 14. The hypertrophic chondrocyte marker COL10A1was significantly increased in nSi (*$P < 0.05$) and ion (Si, Mg, and Li; **$P < 0.01$) samples compared with a negative control. Overall, these results support our hypothesis that nSi and its ionic dissolution products cause endochondral differentiation in hMSCs.

Likewise, matrix mineralization or calcium deposition was monitored after 21 days (Fig. 2D and fig. S1). Quantification of mineralization revealed that treatment with Si (***$P < 0.001$), the combination of ions (**$P < 0.01$), and nSi (*$P < 0.05$) significantly increased matrix mineralization compared with the untreated control. Similarly, Si treatment resulted in significantly higher production of mineralized ECM compared with Li and Mg treatment (***$P < 0.001$). While Si ions alone have a significant effect on hMSC differentiation, this could be attributed to the high concentration of Si treatment, which mirrors the concentrations discerned to be released from nSi. In comparison, Li and Mg have lower treatment concentrations, because these ions are not released at high concentrations from nSi as previously discerned through ICP-MS after 21 days at pH 5.5. In addition, these ion concentrations were added externally to hMSCs compared with the release of ions from the nSi that occurs in the endosome. The local concentration of released Li and Mg within the cell most likely has a greater effect than external addition of the low concentrations of Li or Mg. Overall, treatment with the combination of ions resulted in statistically similar ALP production and matrix mineralization compared with nSi.

Nanosilicates and its dissolution products trigger lineage-specific gene expression program

To investigate the role of nSi and its ionic dissolution products in endochondral differentiation, we performed whole-transcriptome sequencing after 21 days of hMSC samples treated with nSi, individual ions (Si, Mg, or Li), or the combination of ions (Si, Mg, and Li). This study was performed in osteoconductive media (containing β-glycerophosphate and L-ascorbic acid), which did not contain any additional osteoinductive agents such as dexamethasone or recombinant human BMP-2. This approach was used to determine whether nSi or individual ionic dissolution products themselves initiate endochondral differentiation. After 21 days of treatment with individual ion concentrations selected from the ICP-MS data [~31% (4.49 ± 1.98 μg/ml) Si, ~49% (0.067 ± 0.03 μg/ml) Li, and ~18% (1.59 ± 0.85 μg/ml) Mg], the total mRNA was extracted for RNA-seq. RNA-seq was performed, and transcripts were aligned to the human reference genome (hg38). The gene expression levels for every gene in every sample were normalized to obtain fragments per kilobase of transcript per million reads (FPKM). The correlation of expressed gene levels (FPKM) for replicate samples subjected to different conditions indicated a high degree of consistency ($r > 0.95$). Each treatment group was compared to an untreated hMSC control group to obtain differentially expressed genes (DEGs; P-adj < 0.01; see Methods in the Supplementary Materials and data file S1). To obtain DEGs, generalized linear models (GLMs) were used (*38*). The total number of distinct DEGs across all samples was 6340.

Principal components analysis (PCA) of the gene expression profiles revealed the robustness and limited variation between sample replicates (Fig. 3A). DEGs revealed a significant change in gene expression between ion and nSi treatment conditions compared with the untreated hMSCs (Fig. 3B and data file S1). Specifically, hMSCs treated with nSi, Si, Mg, Li, and the combination of all ions (Si, Mg, and Li) showed a significant [Benjamini-Hochberg false discovery rate (FDR); P-adj < 0.01] change in their gene expression profile: 634, 1661, 4000, 3311, and 3685 genes, respectively (Fig. 3C). A number of DEGs were common between the five treatment groups (nSi, Si, Li, Mg, and the combination of all individual ions; fig. S2), but only 113 were detected as DEGs by all five treatments. Furthermore, hMSCs treated with individual ions exhibited a large difference in the number of DEGs, more so than that of nSi.

We next performed gene ontology (GO) pathway enrichment analysis of DEGs to identify pathways that are perturbed by nSi and ion dissolution products. To do this, we used the Bioconductor package GOstats to perform hypergeometric-based tests and generate significant ontology terms related to biological processes. GO terms related to known biological processes and biochemical pathways associated with osteogenesis and endochondral ossification were identified (P-adj < 0.05; Fig. 3D, fig. S3, and data file S2). Key signaling pathways regulating chondrogenesis and endochondral skeleton development (*30*) such as mitogen-activated protein kinase (MAPK), BMP, canonical Wingless-related integration site (cWnt), notch, TGF-β/SMAD, hypoxia (HIF), FGFs, and hedgehog (Shh and Ihh) have been identified. Nanosilicates influenced pathways related to MAPK, BMP, TGF-β/SMAD, FGF, and Ihh. The combination of individual ions had a similar effect but did not regulate GO terms related to notch or Ihh pathways. For individual ions, regulation of key pathways in endochondral development varied. Si perturbed processes related to MAPK, BMP, cWnt, Notch, hypoxia, FGF, and hedgehog (Ihh and Shh), while Mg showed GO terms related to MAPK, Notch, TGF-β/SMAD, hypoxia, and FGF. Li showed the least regulation of key endochondral pathways, as it did not perturb BMP, cWnt, TGF-β/SMAD, or notch signaling. These results suggest that the individual ions such as Si and Mg exhibit strong effect on endochondral differentiation.

To validate the effect of ionic dissolution products on some of the key signaling pathways, we treated hMSCs with Si, Mg, and Li for 14 days in the presence and absence of cWnt inhibitor (10 μM cardamonin; Cayman Chemicals) and MAPK inhibitor (5 μM PD185342; Abcam). Both cWnt signaling (*39*, *40*) and MAPK/ERK signaling (*41*) are implicated in osteogenesis and are highlighted in our GO analysis. Western blot was performed on day 14 to evaluate the effect on ALP production (fig. S4). MAPK inhibition seemed to have the strongest effect on ALP production. However, addition of cWnt inhibitor also decreased the expression of ALP in Si, Mg, and Li samples. Biochemical pathways in osteochondral differentiation often act synergistically, as both cWnt and ERK pathways can be activated by upstream signaling cascades such as BMP/TGF-β (*30*). These results indicate that ions may be involved in regulating more than one signaling pathway to regulate gene expression.

The reduce and visualize gene ontology (REVIGO) GO-term slimming platform was used to extract highly enriched, consolidated terms. GO analysis revealed that nSi regulated processes associated with the ECM and endochondral ossification, suggesting that extracellular changes related to endochondral differentiation may be occurring (Fig. 3E), specifically extracellular structure organization (GO:0043062; $P < 1.6 \times 10^{-53}$) and collagen metabolic processes (GO:003296; $P < 8.1 \times 10^{-21}$). Therefore, nSi not only regulate key biochemical pathways but also direct hMSC ECM organization. These results support our earlier study that highlight the fact that

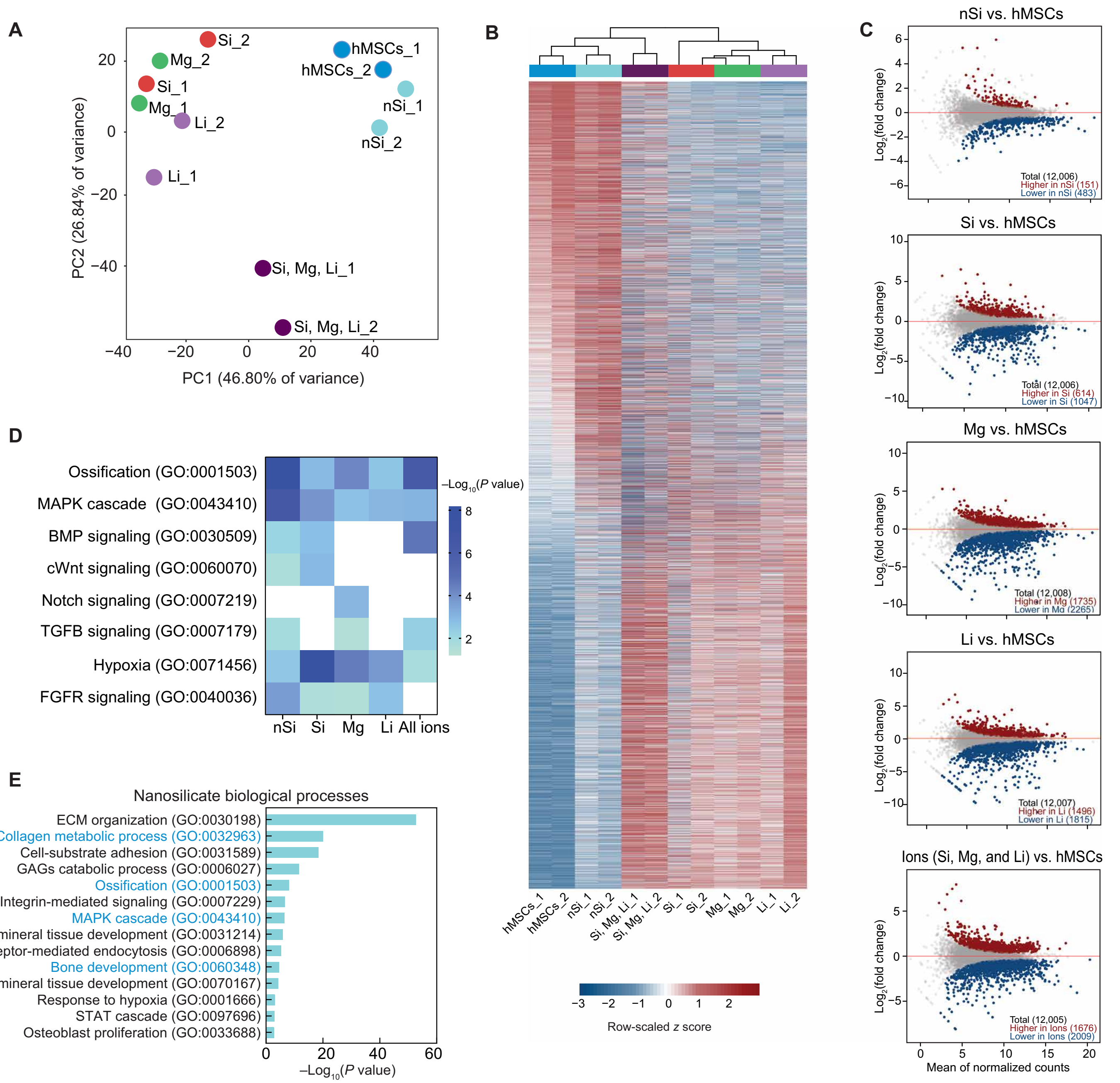

Fig. 3. Nanosilicate and its ionic dissolution products drive transcriptome dynamics. (**A**) Principal component analysis (PCA) of hMSC samples treated with nanosilicates (nSi) and its ionic dissolution products (Si, Mg, Li, and all ions) based on mRNA expression obtained from RNA-seq ($n = 2$, technical replicates). Cells without nanomaterials are used as control (hMSCs). The PCA was done on the mRNA expression (log_2FPKM) of 20% of the most variable genes across all samples ($n = 2362$). (**B**) Hierarchical clustering of hMSC samples treated with nSi, Si, Mg, Li, and all ions based on mRNA expression obtained from RNA-seq. The heatmap shows the differentially expressed genes (DEGs) (log_2FPKM of DEGs; P-adj < 0.05) across all treatment groups compared with control hMSC samples (red, up-regulated; blue, down-regulated). The total number of distinct DEGs across all samples is 6340. (**C**) Difference in gene expression [log_2(fold change)] between hMSC samples treated with nSi, Si, Mg, Li, or all ions. Genes with significantly high expression are shown in red (P-adj < 0.05), while genes with significantly low expression (P-adj < 0.05) are shown in blue. Gray denotes genes that do not exhibit significantly differences. (**D**) The effects of nanosilicates and its ionic dissolution products on key GO terms associated with endochondral ossification and related pathways are shown ($P < 0.05$). Color intensity is associated with increasing $-log_{10}$(P value) (dark blue, greater significance; teal, less significance). (**E**) GO enrichment analysis showed nanosilicates regulate terms associated with ossification (GO:0001503) and extracellular structure organization (GO:0043062) (day 21).

biophysical contribution of nSi via interaction with cell surface receptor can lead to cellular stress, which induces osteogenic differentiation of hMSCs (*19*). Our current study highlights that individual ionic dissolution products and their combination can influence stem cell differentiation, suggesting that intracellular signaling via release of ions may also contribute to the bioactivity of nSi. Hence. it is expected that both biophysical and biochemical characteristics of nSi are uniquely responsible for innate osteoinductivity of nSi.

Individual ion dissolution products contribute to the bioactivity of nSi

Inorganic ions have been implicated to regulate cellular process and potentially control/direct stem cell differentiation. For example, Si contributes to collagen fibril production (*7*, *14*) and regulates cWnt/Shh signaling (*5*). Mg plays a role in promoting osteogenesis (*15*), along with activation of key growth factors involved in this process such as FGF2 and vascular endothelial growth factor (VEGF) (*42*). In addition, Li has been shown to control Wnt signaling by inhibition of GSK3b and thus promotes osteogenesis (*18*, *40*). From the literature and our GO analysis, it is expected that ion dissolution products (Si, Mg, and Li) can induce endochondral differentiation and ossification through multiple mechanisms (Fig. 4A).

Ours results revealed that Si perturbed biological processes for extracellular structure organization (GO:0043062; P-adj < 1.7×10^{-5}) and collagen metabolic processes (GO:003296; P-adj < 1.1×10^{-6}) (Fig. 4B), supporting the role of Si in collagen fibril production (*14*). Furthermore, GO analysis suggested that Si-treated hMSCs are enriched in GO terms related to activation of BMP signaling (GO:0030509; P-adj < 0.0017), cWnt signaling (GO:0060070; P-adj < 0.0012), and positive regulation of MAPK cascades (GO:0043410; P-adj < 0.001) (Fig. 4C). Si treatment also resulted in up-regulation of osteospecific transcription factor *RUNX2* [$\log_2$ (fold change) = 1.23], which was a key contributor to BMP signaling (GO:0030509) (Fig. 4D and fig. S5). BMP signaling results in the subsequent activation of cWnt signaling and MAPK cascades to control the production of osteospecific glycoproteins (*43*). We also observed that Si up-regulates catenin β1 (*CTNNB1*) (β-catenin), which is an important component of the cWnt pathway. Furthermore, Si up-regulated BSP [integrin binding sialoprotein (*IBSP*)] at day 21 [$\log_2$ (fold change) = 2.44], suggesting its ability to facilitate ossification and glycoprotein production. During endochondral ossification, mineralizing hypertrophic chondrocytes promote production of *IBSP* (*44*). Overall, these results suggest that Si contributes to endochondral ossification and promotes chondroblastic to osteoblastic transdifferentiation.

In addition to Si, Mg also was shown to play a key role in inducing osteogenic differentiation of hMSCs. Mg down-regulated common chondrogenic markers such as *ACAN* and *COMP*, but the hypertrophic chondrocyte marker *COL10A1* is up-regulated in Mg-treated hMSCs. Up-regulation of *COL10A1* indicates that Mg-treated hMSCs are transitioned toward hypertrophic chondrocytes. Simultaneous up-regulation of the key osteogenic transcription factor *RUNX2* supports that hypertrophic chondrocyte may begin to transition toward preosteoblast with Mg addition (Fig. 4D). *FGFR2*, a receptor for FGFs, is up-regulated in Mg-treated samples, suggesting that the FGF signaling pathway may be involved in the observed ossification. To confirm the role of Mg in endochondral differentiation, we performed GO analysis. Mg-treated hMSCs showed significant enrichment of GO terms related to ossification (GO:0001503; P-adj < 6.1×10^{-5}) and MAPK cascade (GO:0043410; P-adj < 0.0020) (fig. S6). Earlier studies have implicated the role of Mg in promoting osteogenesis by activating FGF signaling and MAPK cascade (*45*). Mg up-regulated the transcript of *FGFR2*, which was found up-regulated in the MAPK cascade (GO:0043410), suggesting a relationship between the two pathways (fig. S7). Extracellular Mg can enter through solute channels such as TRPM7 or MagT1, which causes subsequent FGF2 expression (*42*). This is further supported by GO analysis, which highlighted the role of Mg in FGF signaling (GO:0044344 cellular response to FGF stimulus; P-adj < 0.0011).

Earlier studies have shown that Li can induce osteogenic differentiation by activating cWnt signaling (*5*, *40*) through inhibition of GSK3b (*16*). As Li inhibits GSK3b by directly interacting with GSK3b (*46*), effect of Li on *GSK3b* mRNA transcripts was not expected. On the basis of our in vitro osteogenic assays, Li treatment was able to induce matrix mineralization (Fig. 2A). This was further supported by the RNA-seq data, which support that Li regulates ossification GO term (GO:0001503; P-adj < 0.0024) (fig. S6). We observed that Li significantly regulated the FGFR signaling pathway (GO:0040036; P-adj < 0.0020), and key genes in these pathways such as *RUNX2* and *FGFR2* were up-regulated [$\log_2$ (fold change) of 0.65 and 0.96, respectively] (Fig. 4D). Thus, it is expected that Li might be promoting osteogenesis, but to a lesser extent compared with Si.

Nanosilicate degradation products control transcriptomic changes in hMSCs

With large expression datasets, there is a potential risk of missing certain perturbed pathways, particularly if genes of interest do not fall below the significance cutoff. Thus, to overcome this challenge and observe the dynamics of all expressed genes, we used gene set enrichment analysis (GSEA). GSEA was used to determine whether an a priori defined set of genes exhibited statistically significant, concordant differences between our treated and untreated hMSCs. To obtain GSEA results, we performed enrichment of curated gene sets in preranked gene lists (see the Supplementary Materials). Enriched gene sets with FDR adjusted $P < 0.1$ were considered in our analysis. The node generation cutoff for this network was set at an FDR adjusted $P < 0.1$ and an edge weight (similarity coefficient) >0.375.

GSEA analysis of all samples and our enrichment analysis suggested that treatment with Si, the combination of all individual ions, or nSi had the ability to regulate processes associated with the ECM, signaling pathways linked to growth factors, and integrin binding, among other responses (Fig. 5A), which corroborates the data found in our previously discussed GO analysis. Nanosilicates, the combination of all individual ions, and Si regulated processes within the gene set group including the *Naba* gene set *Collagens*, and the *Reactome* gene sets *Degradation of the Extracellular Matrix* and *Extracellular Matrix Organization*. Nanosilicates and the combination of individual ions regulated gene sets in the groups containing the *PID* gene set *Integrin Pathway*, the Kyoto Encyclopedia of Genes and Genomes (*KEGG*) gene set *Focal Adhesion*, and the *Reactome* gene set *Glycosaminoglycan Metabolism*. These processes contribute to ECM regulation, cell attachment to the ECM, and extracellular glycoprotein cellular interactions. Silicon has been suggested to interact with prolyl hydroxylase, which catalyzes type 1 collagen production (*14*, *47*). Furthermore, ECM stabilization may be assisted by silicon presence, as silicon is necessary for the binding of proteoglycans to the collagen matrix (*48*). The observation of these ECM changes in silicon, all ion samples, and nSi suggests that intracellular release of silicon may be the mechanism by which nSi promote ECM changes.

As GO analysis does not provide insight into positive or negative process regulation, GSEA analysis showed us that many ECM-related gene sets perturbed by nSi and all ion-treated samples exhibited negative normalized enrichment scores (NES). This score is the primary statistic used to observe gene set correlation and accounts for differences in gene set sizes and thus can be compared across gene set analysis results. A negative NES indicates that the most highly

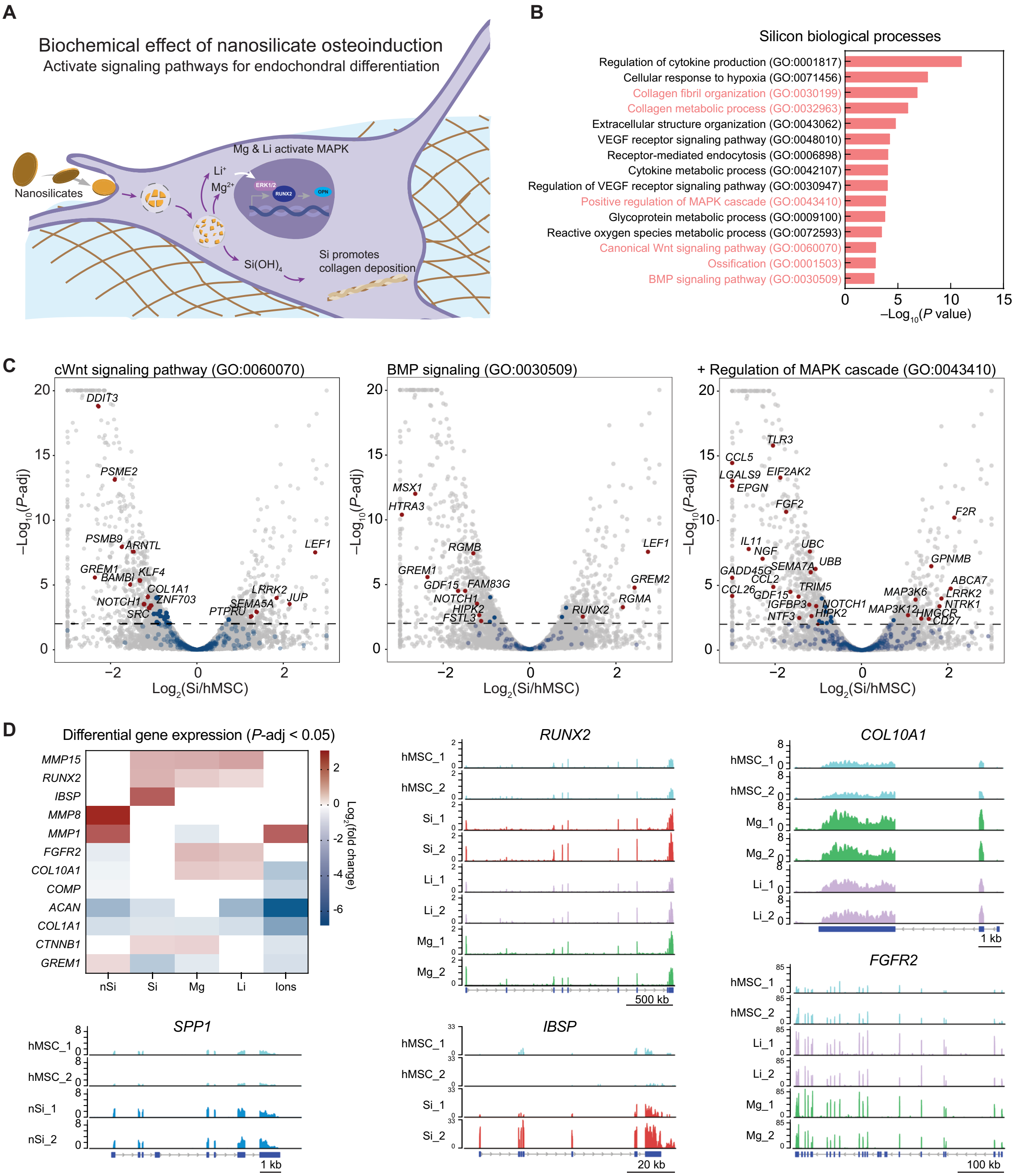

Fig. 4. Individual ions contribute to the innate bioactivity of nanosilicates. (**A**) Schematic depicting the potential role of Si, Li, and Mg in regulating endochondral differentiation. (**B**) GO enrichment analysis showed that Si regulates processes associated with the extracellular matrix such as collagen metabolic processes (GO:0032963) and collagen fibril organization (GO:0030199). (**C**) A volcano plot showing DEGs for BMP signaling (GO:0030509), cWnt signaling (GO:0060070), and MAPK (GO:0043410). Gray, all of the expressed genes; blue, genes associated with the GO term with no significant change in expression; and red, genes associated with the GO term that show significantly different expression. (**D**) DEGs associated with endochondral differentiation showing the effect of nSi, Si, Li, and Mg [$\log_2$(fold change)] (red, up-regulated; blue, down-regulated). RNA-seq tracks showing normalized mRNA expression [aligned reads normalized by total library size—transcript per million (TPM)] at the genomic locus of runt-related transcription factor-2 (*RUNX2*), type 10 collagen (*COL10A1*), secreted phosphoprotein 1 (*SPP1*), integrin binding sialoprotein (*IBSP*), and fibroblast growth factor receptor 2 (*FGFR2*).

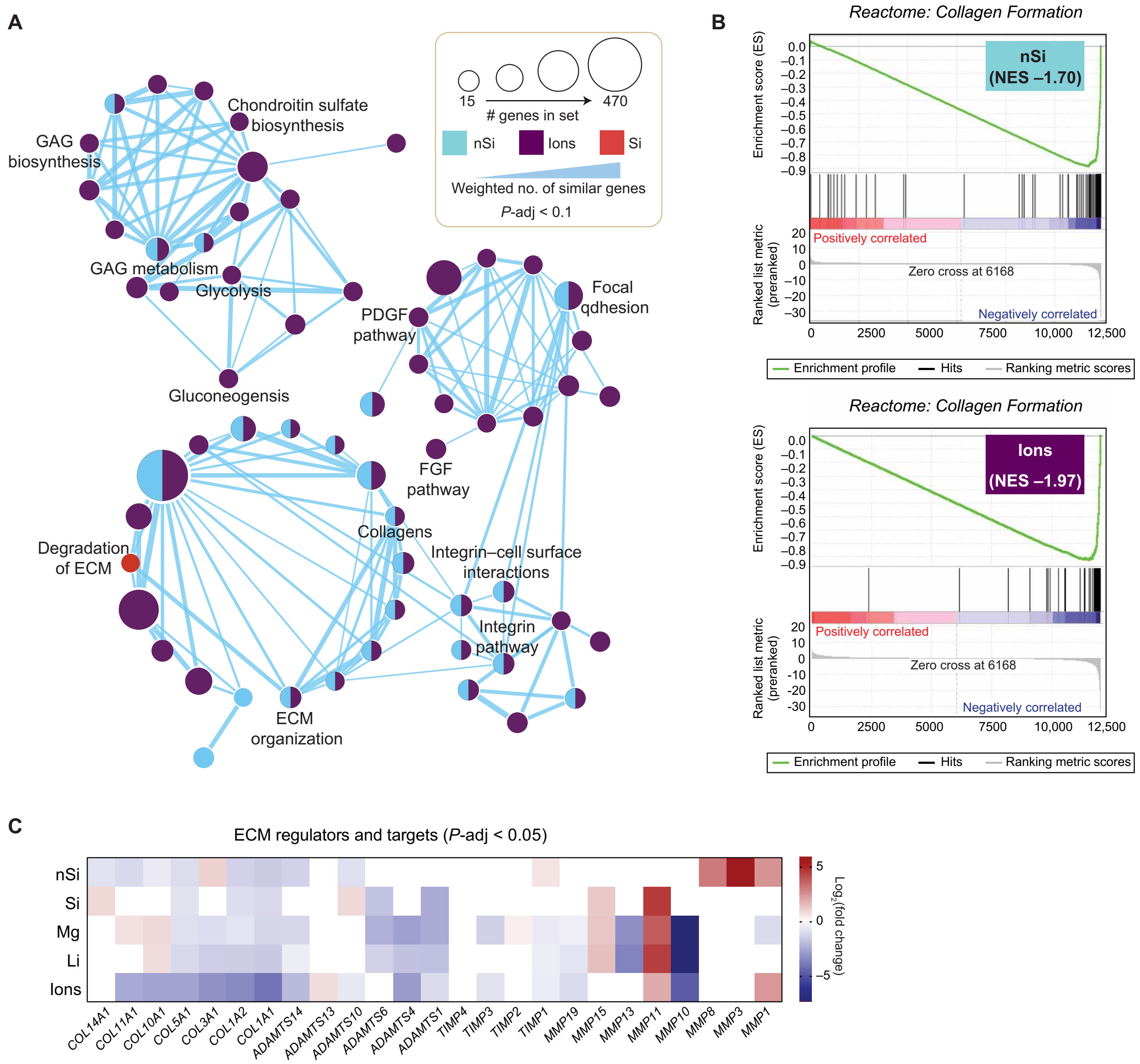

Fig. 5. Gene set enrichment analysis (GSEA) yields insights about the effect of nanosilicate and silicon on endochondral ossification and matrix remodeling. (**A**) GSEA graph displaying interconnected gene sets of hMSC treated with nanosilicates and its ionic dissolution products. The graph was generated in Cytoscape with Enrichment Map using enriched gene sets *P*-adj < 0.1 and an edge similarity coefficient >0.375. The effects of nSi and ions on extracellular matrix organization, glycoproteins, and proteoglycans are shown. (**B**) GSEA enrichment results show normalized enrichment scores (NES) for *Reactome: Collagen Formation* for nSi and ion treatment. The vertical black lines ("bar code") represent the projection onto the ranked gene list of individual genes of the gene set. The horizontal bar in graded color from red (left) to blue (right) represents the gene list ranked from up-regulated on the left to down-regulated on the right. (**C**) Heatmap of DEG (*P*-adj < 0.05), $\log_2$(fold change) expression of genes associated with matrix remodeling including matrix metalloproteinases (MMPs), tissue inhibitors of metalloproteinases (TIMPs), a disintegrin and metalloproteinase with thrombospondin motifs (ADAMTSs), and collagens identified through analysis of nSi transcriptomic data (red, up-regulated; blue, down-regulated). MMP transcripts are most readily positively up-regulated by nSi and ions, including MMP1, MMP3, MMP8, MMP11, and MMP15. Other matrix remodeling enzymes such as ADAMTSs and TIMPs are mostly down-regulated by nSi or ions. Last, collagens are mostly down-regulated, apart from *COL10A1* (up-regulated by Mg and Li), *COL11A1* (up-regulated by Mg), and *COL14A1* (up-regulated by Si).

significant of the enriched genes that correlate to an a priori gene set have a negative fold change. Of note, the *Reactome* gene set *Collagen Formation* (*P*-adj < 0.002) was significantly negatively correlated in nSi, with an NES −1.68, suggesting that further collagen formation was not occurring (Fig. 5B). Similarly, the combination of all ions showed a significant negative correlation for the *Reactome* gene set *Collagen Formation* (*P*-adj < 0.1) with an NES −1.97 (Fig. 5C). Paired with the GSEA data for both nSi and all ion treated samples, these data suggested that there is significant regulation of the ECM occurring at day 21.

As observed through the GO analysis, Si and nSi regulated biological processes controlling ECM and collagen metabolism at day 21. This information is corroborated by GSEA data. Furthermore, at day 21, Si positively regulated the *Reactome* gene set *Degradation of Extracellular Matrix* (P-adj < 0.05, NES = 1.85), which correlated highly significant matrix metalloproteinase (MMPs) 11 and 15 as core enrichments (fig. S8). Core enrichments contribute most to the GSEA enrichment score result. The activation of ECM degradation observed in Si-treated samples suggested that an underlying process of nSi and a combination of individual ion dissolution product treatment may also include the degradation of the ECM. At later time points of endochondral ossification, the cartilaginous template is degraded during ossification (*49*). To do this, MMPs activate to remodel the ECM.

Certain MMPs have been categorized as "collagenases," of which nSi or ion treatment up-regulated three at day 21; *MMP1*, *MMP8*, and *MMP15* (*50*). Although the *Reactome* gene set *Degradation of the Extracellular Matrix* was not significantly regulated by nSi, nSi up-regulates the most MMPs (*MMP1*, *MMP3*, and *MMP8*) of all samples (Fig. 5D). MMP1 (collagenase) and MMP3 (stromelysin) are known to be highly expressed in endochondral ossification (*49*). In addition, ion-treated samples up-regulated MMP11 and MMP15. MMP11 (stromelysin-3) has been found to suppress adipogenesis (*51*) and is secreted during active remodeling in tissues undergoing embryonic development, wound healing, and tumorigenicity (*52*). MMP15 (MT2-MMP) is a membrane bound that is a known collagenase but has a 1:100 decreased effect on type 1 collagen degradation compared with that of MT1-MMP (MMP14) (*53*). However, hMSC culture stability is maintained to day 30 in three-dimensional (3D) hMSC spheroid cultures with the addition of nSi treatments (fig. S9). Therefore, the impact of MMP activation is one associated more with remodeling, which is an expected response during endochondral ossification.

Our study demonstrates that inorganic 2D nSi dissociate into bioactive ions (Li^{+}, Mg^{2+}, and Si^{4+}) at physiological pH and stimulate endochondral differentiation. Ionic dissolution products of inorganic biomaterials can provide unique biomolecular signaling for tissue-specific differentiation. We use whole-transcriptome sequencing (RNA-seq) to characterize the contribution of nSi and its ionic dissolution products on endochondral differentiation. Our study highlights the role of ions in modulating transcriptome dynamics of stem cells by regulating lineage-specific gene expression patterns. Specifically, we have identified that Si promoted matrix mineralization, controlled collagen metabolic processes, and activated osteogenic pathways such as BMP and canonical Wnt at day 21. Furthermore, Mg and Li treatment after 21 days supported the deposition of mineralized matrix and led to the activation of processes controlling MAPK cascades as well as FGFR pathways that contribute to osteogenesis. This work delineates the contribution of individual ionic dissolution products to endochondral differentiation caused by inorganic nSi. Overall, this work paves the way for leveraging biochemical characteristics of inorganic biomaterials to direct cellular processes and promote in situ tissue regeneration.

MATERIALS AND METHODS

Nanosilicate degradation in physiologically relevant pH

The release of minerals from nSi at physiologically relevant pH was monitored using ICP-MS–Elemental Analysis (PerkinElmer NexION 300D). Nanosilicates (Laponite-XLG, BYK Inc., USA) were dispersed in distilled water of various pH values (5.5, 7.4, and 10) and dialyzed against the same pH water over a period of 30 days at room temperature. At various time points (0.125, 1, 3, 7, and 21 days), half of the dialysis water was collected and replaced with fresh water. The collected dialysis water was then diluted into a 1% nitric acid solution for ICP-MS analysis, in which the concentrations of Li, Mg, and Si were determined.

Evaluation of nSi degradation products on hMSC metabolic activity

All experiments were performed with hMSCs passage 5 or lower, and cells were cultured in normal media [α-modified minimal essential media (AMEM); HyClone], 16.5% fetal bovine serum (FBS; Atlanta Biologicals), and 1% penicillin/streptomycin (Gibco), unless otherwise stated. Mineral solutions were prepared using lithium chloride (LiCl), magnesium sulfate ($MgSO_4$), and sodium silicate (Na_2O_3Si) with concentrations between 0 and 10 mg/ml. hMSCs were seeded in 96-well plates at a density of 10,000 cells/cm^2 and after 24 hours were subjected to various mineral concentrations for an additional 24 hours. Minerals were then removed, and an MTT assay (the American Type Culture Collection) was performed according to the manufacturer's protocol. In addition, once desired concentrations were determined, an alamarBlue assay (Thermo Fisher Scientific) was performed at 1, 3, 7, and 14 days to quantify metabolic activity. For both MTT and alamarBlue, control groups consisted of untreated cells (negative) and cells treated with nSi (positive).

Evaluation of nSi degradation products on hMSC osteogenic differentiation

For osteogenic differentiation studies, hMSCs were similarly seeded in 96-well plates at a density of 4000 cells/cm^2. After 24 hours, cells were treated with osteoconductive media [normal growth media supplemented with 10 mM β-glycerophosphate (Sigma-Aldrich) and 50 μM ascorbic acid (BDH Chemicals)] and the various ion concentrations (4.49 μg/ml Si, 0.067 μg/ml Li, and 1.59 μg/ml Mg) for an additional 48 hours; similar controls were used. After 48 hours, minerals and nSi were removed, and cells were continuously treated with osteoconductive media and ions for the remainder of the differentiation study. To analyze osteogenic differentiation, ALP staining and kinetic activity were monitored along with matrix mineralization and quantification. First, hMSCs were fixed with 2.5% glutaraldehyde for 15 to 20 min. At days 7 and 14, ALP staining was done using NBT/BCIP 1-Step solution (Thermo Fisher Scientific) for 30 to 60 min at room temperature. For quantification of the ALP activity, hMSCs were incubated with ALP yellow (SensoLyte pNPP ALP assay kit, AnaSpec). Using an automated plate reader (Tecan), ALP activity as a function of pNPP metabolism (ΔOD405) was measured, and activity was normalized to DNA (PicoGreen, Thermo Fisher Scientific). After 14 and 21 days, Alizarin Red staining (ARS; Electron Microscopy Sciences) was performed. The bound ARS, which is proportional to calcified matrix, was quantified by dissolution in acetic acid (10%), neutralized by ammonium hydroxide (10%), and then measured in an automated plate reader (ΔOD405; Tecan). Both ALP and mineralized matrix were visualized with a stereomicroscope (Zeiss).

Western blot evaluation of osteospecific protein production caused by nSi degradation products

For Western blot, proteins were isolated after 14 days with Laemmli buffer (0.2% bromophenol blue, 20% glycerol, 100 mM tris-HCl,

10% 2-mercaptoethanol, and 4% SDS). Protein samples were separated via gel electrophoresis (Invitrogen, Mini Gel Tank), and gels were then transferred (Invitrogen, iBlot 2) to a nitrocellulose membrane per the manufacturer's protocol. Membranes were blocked with 5% bovine serum albumin (BSA) in PBST (1× PBS with 0.1% Tween 20) for 30 min before antibody staining. β-actin, alkaline phosphatase (ALPL), OCN, OPN, and type 1 collagen (COL1A1) primary antibodies were purchased from Thermo Fisher Scientific; COL10A1, ACAN, and COMP were purchased from ABclonal, secondary horseradish peroxidase (HRP)–conjugated antibodies were purchased from Boster Bio; and incubation was performed following the manufacturer's protocols. Membranes were developed (SuperSignal West Pico PLUS Chemiluminescent Substrate, Thermo Fisher Scientific) and imaged using LI-COR 3600 C-Digit Blot Scanner. LI-COR software or ImageJ analysis was used to quantify protein bands. Restoration and reblocking with 5% BSA in PBST of the membranes were then done for further protein analysis.

Western blot evaluation of osteospecific pathway inhibition

Cells that were cultured with proteins were isolated after 14 days with radioimmunoprecipitation assay lysis buffer (Thermo Fisher Scientific). Canonical Wnt inhibition was performed by treating hMSCs with 10 μM cardamonin (Cayman Chemical) every media change (3 to 4 days). MEK1/2 was inhibited by treating with PD185342 at 5 μM (Abcam) every media change. Protein samples were separated via gel electrophoresis (Invitrogen, Mini Gel Tank), and gels were then transferred (Invitrogen, iBlot 2) to a nitrocellulose membrane per the manufacturer's protocol. Membranes were blocked with 5% BSA in PBST for 30 min before antibody staining. β-actin, ALPL, type 1 collagen (COL1A1), and RUNX2 primary antibodies were purchased from Thermo Fisher Scientific; secondary HRP-conjugated antibodies were purchased from Boster Bio; and incubation was performed following the manufacturer's protocols. Membranes were developed (SuperSignal West Pico PLUS Chemiluminescent Substrate, Thermo Fisher Scientific) and imaged using LI-COR 3600 C-Digit Blot Scanner. ImageJ analysis was used to quantify protein bands. Restoration and reblocking with 5% BSA in PBST of the membranes were then done for further protein analysis.

RNA-seq sample preparation

For mRNA extraction, cells were cultured until 65% confluent and subjected to two different media compositions for 21 days. One set of cells was cultured with osteoconductive media as a negative control (two replicates); another set was treated with nSi {Laponite XLG $Na^{+}_{0.7}[(Mg_{5.5}Li_{0.3}Si_8O_{20}(OH)_4]^{-}_{0.7}$} (100 μg/ml) in osteoconductive media (two replicates); another set was treated with Si (4.49 μg/ml) in osteoconductive media (two replicates); another was treated with Li (0.067 μg/ml) in osteoconductive media (two replicates); another was treated with magnesium (1.59 μg/ml) in osteoconductive media (two replicates); and one set was cultured with a combination of all individual ions (4.49 μg/ml Si, 0.067 μg/ml Li, and 1.59 μg/ml Mg), after which media and treatments were performed every 3 to 4 days until day 21. On the 21st day, cells were washed with PBS and pelleted. RNA was isolated and collected via a Zymogen, High Purity RNA Isolation kit following the manufacturer's protocol. Quality of nucleic acids (1 μg) was evaluated using spectrometer absorbance ratios between 280 and 260 nm around 2.0.

RNA-seq processing

RNA samples were analyzed via a high-output NovaSeq platform with TruSeq RNA sample preparation and single-end read length of 125 bases (Charlie Johnson, Genomics and Bioinformatics Service, Texas A&M AgriLife Research, College Station, TX). Sequence reads were trimmed and aligned to the human genome (hg38) using an RNA-seq aligner, Spliced Transcripts Alignment to a Reference (STAR) (*54*). STAR is an algorithm that aligns RNA-seq reads generated from spliced RNA. The Reference sequence (RefSeq) genome annotation of the human genome (hg38; GRCh37 Genome Reference Consortium Human Reference 37) was obtained from the University of California, Santa Cruz, genome browser and was used to find the gene definition. For the negative control group (hMSCs in osteoconductive media), 23,064,842 (20,783,935 uniquely mapped) and 33,923,628 (30,981,658 uniquely mapped) reads were aligned to the genome for the two replicates. For the nSi treatment group, 17,700,673 (16,256,612 uniquely mapped) and 26,835,751 (24,998,082 uniquely mapped) reads were successfully aligned to the genome for the two replicates. For the silicon treatment group, 31,106,835 (28,281,969 uniquely mapped) and 25,227,190 (23,346,719 uniquely mapped) reads successfully aligned to the genome for the two replicates. For the Li treatment group, 37,028,855 (33,824,437 uniquely mapped) and 26,033,891 (23,758,531 uniquely mapped) reads successfully aligned to the genome for the two replicates. For the magnesium treatment group, 33,235,878 (30,983,098 uniquely mapped) and 25,183,849 (23,651,840 uniquely mapped) reads successfully aligned to the genome for the two replicates. For the combination of all individual ion dissolution product treatment group, 27,646,481 (25,461,405 uniquely mapped) and 24,116,483 (22,400,212 uniquely mapped) reads successfully aligned to the genome for the two replicates. In further analysis, only uniquely mapped reads were used.

Identifying DEGs

Gene models were generated using the Bioconductor package GenomicFeatures in R (*55*). Gene expression was determined by counting the uniquely mapped reads associated with coding exons and normalized by gene length in reads per kilobase per million (RPKM). This measure was used only to filter expressed genes. Greater than 1 RPKM is a reasonable cutoff to remove genes with minimal to no expression. Genes greater than 1 RPKM were considered to be expressed in any condition if present in both replicates. Genes expressed in only one condition were considered differentially expressed. DEGs identified by GLMs were modeled as a negative binomial distribution (*56*). DESeq2, a Bioconductor package, was used to do this (*38*). R was used to perform all analyses.

GO enrichment analysis

We performed functional annotation enrichment on the identified DEGs using the Bioconductor (*57*) package GoStats to perform GO enrichment analysis within the biological processes ontology and generate associated volcano plots (*58*). GoStats analysis used conditional hypergeometric test of overrepresentation against the annotation package org.Hs.eg.db, with a DEG cutoff of FDR P-adj < 0.05. The Gviz package was also used to generate gene tracks (*59*). REVIGO (*60*) refined the long list of significant GO terms, which decreases functional redundancies and clusters terms based on semantic similarity measures. Included genes in the GO enrichment analysis had an FDR P-adj < 0.05.

Gene set enrichment analysis

To obtain targeted enrichment analyses of our DEGs, we used GSEA analysis using the reference database Molecular Signatures Database (MsigDB) v7.2 as described from previous publications (*61*). Explicitly, ranked lists of DEGs were generated by applying a $-\log_{10}$ transformation to the *P*-adj value, then multiplying by the sign of the calculated $\log_2$ fold change. GSEA was run on the basis of the ranked lists using default settings, which included "collapse" to collapse dataset to gene symbols before analysis and excluded gene set sizes of greater than 500 and less than 15. The ranked lists were used to compare to a priori sets of genes within the c2 curated collection (version 6) of gene sets obtained from the MsigDB (v7.2) during GSEA analysis (https://data.broadinstitute.org/gsea-msigdb/msigdb/release/6.2/c2.all.v6.2.symbols.gmt). The chip platform used was Human_NCBI_Entrez_Gene_ID_MSigDB.v7.2.chip. The top 20 plots were graphed for both positive and negative gene set results. Gene sets with an FDR of less than 10% (FDR < 0.1) were considered significant.

Gene set enrichment map network

GSEA analysis was used to perform gene set enrichment and in conjunction with Cytoscape for gene set networks (*61*). To do so, GSEA software (version 4.1.0) was used to perform enrichment map visualization in conjugation with Cytoscape (version 3.8.0) (*62*). Cytoscape was used to arrange gene set networks generated through the Enrichment Map plug-in (*63*). To do this, the preranked lists, positive and negative enrichment reports, and gene set.gmt files were loaded to build the network with the analysis type set to "GSEA." The "data set edges" option was set to automatic, with the "connectivity" slide bar set to default settings. Gene sets included in the GSEA network analysis had a *P*-adj value (Benjamini-Hochberg FDR) <0.1. Node cutoff was set at an FDR value of <0.1. The "edge cutoff (similarity)" was set to 0.375. Nodes were stylized to show the designated colors of treatment group, which regulate gene sets. Node colors do not represent positive or negative NES. Node size represents the size of gene sets. Edge weight represents the degree of similarity, specifically the number of shared genes between gene sets.

Spheroid culture

hMSCs were grown in 2D to confluence in normal media [AMEM (HyClone), 16.5% FBS (Atlanta Biologicals), and 1% penicillin/streptomycin (Gibco)]. After reaching 80% confluence, the cells were starved with serum-free media for 16 hours to maximize uptake of nanoparticles. This was followed by treatment with nSi for 24 hours. After 24 hours, the 2D culture was converted into 3D spheroids consisting of 10^6 cells per spheroid. Control hMSC spheroids received no additional treatment. Media were replenished every 2 days. After 30 days of culture, spheroids were washed (PBS), fixed with buffered 4% paraformaldehyde, embedded in paraffin, and processed into 10-μm sections. Paraffinized sections were treated sequentially with xylene (three changes) to deparaffinize, and then a series of ethanol: 100 (three changes), 95 (two changes), and 70% (one change), for 2 min each to rehydrate. Sections were then washed for 5 min with DI water. Prepared sections were briefly rinsed with 3% acetic acid (30 s) and then stained. GAG production was evaluated using Alcian Blue (MP Biomedicals), which was prepared by dissolving 1 g of 86× Alcian Blue powder in 3% acetic acid, and then buffered with acetic acid until pH 2.5. Staining was performed for 30 min and then washed with DI water for 2 min to remove excess stain. Von Kossa (Sigma-Aldrich) staining was used to evaluate calcium deposition. In brief, sections were incubated with 1% silver nitrate solution under ultraviolet light for 20 min, washed with DI water, and treated with 5% sodium thiosulfate for 5 min. Counter stain was performed with nuclear fast red. Stained sections were dehydrated in a series of ethanol and cleared in xylene before mounting with Permount Mounting medium and imaging. Images were obtained using a Lionheart FX microscope. Quantification of Von Kossa staining was performed using Lionheart FX software, and quantification of Alcian Blue was performed using ImageJ software. In brief, Alcian Blue images were converted to gray scale, color thresholding was applied over the staining cohort, and areas meeting the color threshold were quantified and normalized to the total area of the spheroid. Values were normalized to the negative control (hMSC spheroids), and an unpaired *t* test was used to perform statistical analysis.

Statistical analysis

Statistical analysis was performed in GraphPad Prism. One-way analysis of variance (ANOVA) coupled with Tukey's post hoc were performed. Unpaired *t* tests were used to perform statistical analysis between the treatment and control samples for Western blot analysis. Plots were graphed as means and SD, and statistical significance is presented as $*P < 0.05$; $**P < 0.01$; $***P < 0.001$.

SUPPLEMENTARY MATERIALS

Supplementary material for this article is available at https://science.org/doi/10.1126/sciadv.abl9404

View/request a protocol for this paper from *Bio-protocol*.

REFERENCES AND NOTES

1. A. K. Gaharwar, I. Singh, A. Khademhosseini, Engineered biomaterials for in situ tissue regeneration. *Nat. Rev.Mater.* **5**, 686–705 (2020).
2. A. Hoppe, N. S. Güldal, A. R. Boccaccini, A review of the biological response to ionic dissolution products from bioactive glasses and glass-ceramics. *Biomaterials* **32**, 2757–2774 (2011).
3. A. M. Brokesh, A. K. Gaharwar, Inorganic biomaterials for regenerative medicine. *ACS Appl. Mater. Interfaces* **12**, 5319–5344 (2020).
4. A. Murali, G. Lokhande, K. A. Deo, A. Brokesh, A. K. Gaharwar, Emerging 2D nanomaterials for biomedical applications. *Mater. Today* **50**, 276–302 (2021).
5. P. Han, C. Wu, Y. Xiao, The effect of silicate ions on proliferation, osteogenic differentiation and cell signalling pathways (WNT and SHH) of bone marrow stromal cells. *Biomater. Sci.* **1**, 379–392 (2013).
6. T. Hu, H. Xu, C. Wang, H. Qin, Z. An, Magnesium enhances the chondrogenic differentiation of mesenchymal stem cells by inhibiting activated macrophage-induced inflammation. *Sci. Rep.* **8**, 3406 (2018).
7. E. M. Carlisle, In vivo requirement for silicon in articular cartilage and connective tissue formation in the chick. *J. Nutr.* **106**, 478–484 (1976).
8. C. Yi, D. Liu, C.-C. Fong, J. Zhang, M. Yang, Gold nanoparticles promote osteogenic differentiation of mesenchymal stem cells through p38 MAPK pathway. *ACS Nano* **4**, 6439–6448 (2010).
9. H. Wang, S. Zhao, J. Zhou, Y. Shen, W. Huang, C. Zhang, M. N. Rahaman, D. Wang, Evaluation of borate bioactive glass scaffolds as a controlled delivery system for copper ions in stimulating osteogenesis and angiogenesis in bone healing. *J. Mater. Chem. B* **2**, 8547–8557 (2014).
10. S. D. Gower-Winter, C. W. Levenson, Zinc in the central nervous system: From molecules to behavior. *Biofactors* **38**, 186–193 (2012).
11. C. Nozaki, A. M. Vergnano, D. Filliol, A.-M. Ouagazzal, A. Le Goff, S. Carvalho, D. Reiss, C. Gaveriaux-Ruff, J. Neyton, P. Paoletti, B. L. Kieffer, Zinc alleviates pain through high-affinity binding to the NMDA receptor NR2A subunit. *Nat. Neurosci.* **14**, 1017–1022 (2011).
12. Y. Qiao, W. Zhang, P. Tian, F. Meng, H. Zhu, X. Jiang, X. Liu, P. K. Chu, Stimulation of bone growth following zinc incorporation into biomaterials. *Biomaterials* **35**, 6882–6897 (2014).
13. E. Mostaed, M. Sikora-Jasinska, J. W. Drelich, M. Vedani, Zinc-based alloys for degradable vascular stent applications. *Acta Biomater.* **71**, 1–23 (2018).
14. D. M. Reffitt, N. Ogston, R. Jugdaohsingh, H. F. J. Cheung, B. A. J. Evans, R. P. H. Thompson, J. Powell, G. N. Hampson, Orthosilicic acid stimulates collagen type 1 synthesis

and osteoblastic differentiation in human osteoblast-like cells in vitro. *Bone* **32**, 127–135 (2003).
15. S. Yoshizawa, A. Brown, A. Barchowsky, C. Sfeir, Magnesium ion stimulation of bone marrow stromal cells enhances osteogenic activity, simulating the effect of magnesium alloy degradation. *Acta Biomater.* **10**, 2834–2842 (2014).
16. F. Zhang, C. J. Phiel, L. Spece, N. Gurvich, P. S. Klein, Inhibitory phosphorylation of glycogen synthase kinase-3 (GSK-3) in response to lithium. Evidence for autoregulation of GSK-3. *J. Biol. Chem.* **278**, 33067–33077 (2003).
17. L. Li, X. Peng, Y. Qin, R. Wang, J. Tang, X. Cui, T. Wang, W. Liu, H. Pan, B. Li, Acceleration of bone regeneration by activating Wnt/β-catenin signalling pathway via lithium released from lithium chloride/calcium phosphate cement in osteoporosis. *Sci. Rep.* **7**, 45204 (2017).
18. C. A. Gregory, A. S. Perry, E. Reyes, A. Conley, W. G. Gunn, D. J. Prockop, Dkk-1-derived synthetic peptides and lithium chloride for the control and recovery of adult stem cells from bone marrow. *J. Biol. Chem.* **280**, 2309–2323 (2005).
19. J. K. Carrow, L. M. Cross, R. W. Reese, M. K. Jaiswal, C. A. Gregory, R. Kaunas, I. Singh, A. K. Gaharwar, Widespread changes in transcriptome profile of human mesenchymal stem cells induced by two-dimensional nanosilicates. *Proc. Natl. Acad. Sci. U.S.A.* **115**, E3905–E3913 (2018).
20. A. K. Gaharwar, L. M. Cross, C. W. Peak, K. Gold, J. K. Carrow, A. Brokesh, K. A. Singh, 2D nanoclay for biomedical applications: Regenerative medicine, therapeutic delivery, and additive manufacturing. *Adv. Mater.* **31**, 1900332 (2019).
21. J. R. Xavier, T. Thakur, P. Desai, M. K. Jaiswal, N. Sears, E. Cosgriff-Hernandez, R. Kaunas, A. K. Gaharwar, Bioactive nanoengineered hydrogels for bone tissue engineering: A growth-factor-free approach. *ACS Nano* **9**, 3109–3118 (2015).
22. J. I. Dawson, R. O. C. Oreffo, Clay: New opportunities for tissue regeneration and biomaterial design. *Adv. Mater.* **25**, 4069–4086 (2013).
23. M. Mousa, N. D. Evans, R. O. C. Oreffo, J. I. Dawson, Clay nanoparticles for regenerative medicine and biomaterial design: A review of clay bioactivity. *Biomaterials* **159**, 204–214 (2018).
24. A. K. Gaharwar, S. M. Mihaila, A. Swami, A. Patel, S. Sant, R. L. Reis, A. P. Marques, M. E. Gomes, A. Khademhosseini, Bioactive silicate nanoplatelets for osteogenic differentiation of human mesenchymal stem cells. *Adv. Mater.* **25**, 3329–3336 (2013).
25. D. W. Thompson, J. T. Butterworth, The nature of laponite and its aqueous dispersions. *J. Colloid Interface Sci.* **151**, 236–243 (1992).
26. S. Jatav, Y. M. Joshi, Chemical stability of Laponite in aqueous media. *Appl. Clay Sci.* **97-98**, 72–77 (2014).
27. P. Aghajanian, S. Mohan, The art of building bone: Emerging role of chondrocyte-to-osteoblast transdifferentiation in endochondral ossification. *Bone Res.* **6**, 19 (2018).
28. S. Provot, E. Schipani, J. Y. Wu, H. Kronenberg, Chapter 6 - Development of the Skeleton, in *Osteoporosis* (*Fourth Edition*), R. Marcus, D. Feldman, D. W. Dempster, M. Luckey, J. A. Cauley, Eds. (Academic Press, 2013), pp. 97–126.
29. R. Marsell, T. A. Einhorn, The biology of fracture healing. *Injury* **42**, 551–555 (2011).
30. J. D. Green, V. Tollemar, M. Dougherty, Z. Yan, L. Yin, J. Ye, Z. Collier, M. K. Mohammed, R. C. Haydon, H. H. Luu, R. Kang, M. J. Lee, S. H. Ho, T.-C. He, L. L. Shi, A. Athiviraham, Multifaceted signaling regulators of chondrogenesis: Implications in cartilage regeneration and tissue engineering. *Genes Dis.* **2**, 307–327 (2015).
31. T. A. Karlsen, R. B. Jakobsen, T. S. Mikkelsen, J. E. Brinchmann, microRNA-140 targets *RALA* and regulates chondrogenic differentiation of human mesenchymal stem cells by translational enhancement of *SOX9* and *ACAN*. *Stem Cells Dev.* **23**, 290–304 (2013).
32. M. B. Goldring, K. Tsuchimochi, K. Ijiri, The control of chondrogenesis. *J. Cell. Biochem.* **97**, 33–44 (2006).
33. W. Tong, R. J. Tower, C. Chen, L. Wang, L. Zhong, Y. Wei, H. Sun, G. Cao, H. Jia, M. Pacifici, E. Koyama, M. Enomoto-Iwamoto, L. Qin, Periarticular mesenchymal progenitors initiate and contribute to secondary ossification center formation during mouse long bone development. *Stem Cells* **37**, 677–689 (2019).
34. G. Shen, The role of type X collagen in facilitating and regulating endochondral ossification of articular cartilage. *Orthod. Craniofac. Res.* **8**, 11–17 (2005).
35. J. Park, M. Gebhardt, S. Golovchenko, F. Perez-Branguli, T. Hattori, C. Hartmann, X. Zhou, B. deCrombrugghe, M. Stock, H. Schneider, K. von der Mark, Dual pathways to endochondral osteoblasts: A novel chondrocyte-derived osteoprogenitor cell identified in hypertrophic cartilage. *Biol. Open* **4**, 608–621 (2015).
36. L. Gerstenfeld, F. Shapiro, Expression of bone-specific genes by hypertrophic chondrocytes: Implications of the complex functions of the hypertrophic chondrocyte during endochondral bone development. *J. Cell. Biochem.* **62**, 1–9 (1996).
37. J. Lian, M. McKee, A. Todd, L. C. Gerstenfeld, Induction of bone-related proteins, osteocalcin and osteopontin, and their matrix ultrastructural localization with development of chondrocyte hypertrophy in vitro. *J. Cell. Biochem.* **52**, 206–219 (1993).
38. M. I. Love, W. Huber, S. Anders, Moderated estimation of fold change and dispersion for RNA-seq data with DESeq2. *Genome Biol.* **15**, 550 (2014).
39. B. H. Clough, S. Zeitouni, U. Krause, C. D. Chaput, L. M. Cross, A. K. Gaharwar, C. A. Gregory, Rapid osteogenic enhancement of stem cells in human bone marrow using a glycogen-synthease-kinase-3-beta inhibitor improves osteogenic efficacy in vitro and in vivo. *Stem Cells Transl. Med.* **7**, 342–353 (2018).
40. P. Clement-Lacroix, M. Ai, F. Morvan, S. Roman-Roman, B. Vayssiere, C. Belleville, K. Estrera, M. L. Warman, R. Baron, G. Rawadi, Lrp5-independent activation of Wnt signaling by lithium chloride increases bone formation and bone mass in mice. *Proc. Natl. Acad. Sci. U.S.A.* **102**, 17406–17411 (2005).
41. M. Li, P. He, Y. Wu, Y. Zhang, H. Xia, Y. Zheng, Y. Han, Stimulatory effects of the degradation products from Mg-Ca-Sr alloy on the osteogenesis through regulating ERK signaling pathway. *Sci. Rep.* **6**, 32323 (2016).
42. D. Zhu, J. You, N. Zhao, H. Xu, Magnesium regulates endothelial barrier functions through TRPM7, MagT1, and S1P1. *Adv. Sci.* **6**, 1901166 (2019).
43. G. Chen, C. Deng, Y.-P. Li, TGF-β and BMP signaling in osteoblast differentiation and bone formation. *Int. J. Biol. Sci.* **8**, 272–288 (2012).
44. X. Qin, Q. Jiang, K. Nagano, T. Moriishi, T. Miyazaki, H. Komori, K. Ito, K. von der Mark, C. Sakane, H. Kaneko, T. Komori, Runx2 is essential for the transdifferentiation of chondrocytes into osteoblasts. *PLOS Genet.* **16**, e1009169 (2020).
45. H. Qi, Y. Liu, L. Wu, S. Ni, J. Sun, J. Xue, Q. Liu, X. Ni, W. Fan, MicroRNA-16, via FGF2 regulation of the ERK/MAPK pathway, is involved in the magnesium-promoted osteogenic differentiation of mesenchymal stem cells. *Oxid. Med. Cell Longev.* **2020**, 3894926 (2020).
46. E. Beurel, S. F. Grieco, R. S. Jope, Glycogen synthase kinase-3 (GSK3): Regulation, actions, and diseases. *Pharmacol. Ther.* **148**, 114–131 (2015).
47. J. D. Birchall, The essentiality of silicon in biology. *Chem. Soc. Rev.* **24**, 351–357 (1995).
48. K. Schwarz, A bound form of silicon in glycosaminoglycans and polyuronides. *Proc. Natl. Acad. Sci. U.S.A.* **70**, 1608–1612 (1973).
49. N. Ortega, D. J. Behonick, Z. Werb, Matrix remodeling during endochondral ossification. *Trends Cell Biol.* **14**, 86–93 (2004).
50. G.-J. Boelen, L. Boute, J. d'Hoop, M. EzEldeen, I. Lambrichts, G. Opdenakker, Matrix metalloproteinases and inhibitors in dentistry. *Clin. Oral Investig.* **23**, 2823–2835 (2019).
51. M.-C. Rio, Chapter 160 - Matrix Metalloproteinase-11/Stromelysin 3, in *Handbook of Proteolytic Enzymes* (*Third Edition*), N. D. Rawlings, G. Salvesen, Eds. (Academic Press, 2013), pp. 779–786.
52. N. Cui, M. Hu, R. A. Khalil, Chapter One - Biochemical and Biological Attributes of Matrix Metalloproteinases, in *Progress in Molecular Biology and Translational Science*, R. A. Khalil, Ed. (Academic Press, 2017), **147**, pp. 1–73.
53. Y. Itoh, Membrane-type matrix metalloproteinases: Their functions and regulations. *Matrix Biol.* **44-46**, 207–223 (2015).
54. A. Dobin, C. A. Davis, F. Schlesinger, J. Drenkow, C. Zaleski, S. Jha, P. Batut, M. Chaisson, T. R. Gingeras, STAR: Ultrafast universal RNA-seq aligner. *Bioinformatics* **29**, 15–21 (2013).
55. M. Lawrence, W. Huber, H. Pagès, P. Aboyoun, M. Carlson, R. Gentleman, M. T. Morgan, V. J. Carey, Software for computing and annotating genomic ranges. *PLoS Comput. Biol.* **9**, e1003118 (2013).
56. S. Anders, W. Huber, Differential expression analysis for sequence count data. *Genome Biol.* **11**, R106 (2010).
57. R. C. Gentleman, V. J. Carey, D. M. Bates, B. Bolstad, M. Dettling, S. Dudoit, B. Ellis, L. Gautier, Y. Ge, J. Gentry, Bioconductor: Open software development for computational biology and bioinformatics. *Genome Biol.* **5**, R80 (2004).
58. S. Falcon, R. Gentleman, Using GOstats to test gene lists for GO term association. *Bioinformatics* **23**, 257–258 (2007).
59. F. Hahne, R. Ivanek, Visualizing Genomic Data Using Gviz and Bioconductor, in *Statistical Genomics: Methods and Protocols*, E. Mathé, S. Davis, Eds. (Springer New York, 2016), pp. 335–351.
60. F. Supek, M. Bošnjak, N. Škunca, T. Šmuc, REVIGO summarizes and visualizes long lists of gene ontology terms. *PLOS ONE* **6**, e21800 (2011).
61. A. Subramanian, P. Tamayo, V. K. Mootha, S. Mukherjee, B. L. Ebert, M. A. Gillette, A. Paulovich, S. L. Pomeroy, T. R. Golub, E. S. Lander, J. P. Mesirov, Gene set enrichment analysis: A knowledge-based approach for interpreting genome-wide expression profiles. *Proc. Natl. Acad. Sci.U.S.A.* **102**, 15545–15550 (2005).
62. P. Shannon, A. Markiel, O. Ozier, N. S. Baliga, J. T. Wang, D. Ramage, N. Amin, B. Schwikowski, T. Ideker, Cytoscape: A software environment for integrated models of biomolecular interaction networks. *Genome Res.* **13**, 2498–2504 (2003).
63. D. Merico, R. Isserlin, O. Stueker, A. Emili, G. D. Bader, Enrichment map: A network-based method for gene-set enrichment visualization and interpretation. *PLOS ONE* **5**, e13984 (2010).

Acknowledgments: We acknowledge help from X. Ding and M. Jaiswal. Some of the images in the article were created with www.Biorender.com. **Funding:** A.K.G. acknowledges financial support from the National Institute of Biomedical Imaging and Bioengineering (NIBIB) of the National Institutes of Health (NIH), Director's New Innovator Award (DP2 EB026265), and

President's Excellence Fund (X-Grants) from Texas A&M University. I.S. and A.K.G. acknowledge financial support from the National Institute of Neurological Disorders and Stroke (R21 NS121945) and the TAMU President's Excellence Fund for Triads for Transformation (T-3, 243677). In addition, I.S. acknowledges Texas A&M University start-up funds (123026). The content is solely the responsibility of the authors and does not necessarily represent the official views of the funding agency. **Author contributions:** A.M.B. and L.M.C. contributed equally and have the right to list their names first. Similarly, I.S. and A.K.G. are co-corresponding authors and have the right to list their names last. A.K.G., I.S., L.M.C., and A.M.B. conceived the original idea of the project. A.K.G. and I.S. provided funds, instruments, and guidance. A.M.B., L.M.C., A.M., and C.R. performed data collection and analysis. I.S. and A.L.K. led the computational analysis and data processing. C.A.G. helped in data interpretation. A.M.B., L.M.C., I.S., and A.K.G. wrote the manuscript. All coauthors commented on the results and participated in the writing and critical revision of the manuscript. **Competing interests:** The authors declare that they have no competing interests. **Data and materials availability:** All data needed to evaluate the conclusions in the paper are present in the paper and/or the Supplementary Materials. RNA-seq data reported here have been deposited in the Gene Expression Omnibus (GEO) database (accession no. GSE193172; www.ncbi.nlm.nih.gov/geo).

Submitted 16 August 2021
Accepted 11 March 2022
Published 27 April 2022
10.1126/sciadv.abl9404
