# Supplementary material for: Dissociation of nanosilicates induces downstream endochondral differentiation gene expression program
Source: Sci Adv. 2022 Apr 27;8(17):eabl9404. doi: 10.1126/sciadv.abl9404 (PMC9045714; doi:10.1126/sciadv.abl9404)
Supplement: Supplementary file 2 — Figs. S1 to S9 [file sciadv.abl9404_sm.pdf]

Science Advances
AAAS

Supplementary Materials for

Dissociation of nanosilicates induces downstream endochondral differentiation gene expression program

Anna M. Brokesh, Lauren M. Cross, Anna L. Kersey, Aparna Murali, Christopher Richter, Carl A. Gregory, Irtisha Singh*, Akhilesh K. Gaharwar*

*Corresponding author. Email: gaharwar@tamu.edu (A.G.); isingh@tamu.edu (I.S.)

Published 27 April 2022, *Sci. Adv.* **8**, eabl9404 (2022)
DOI: 10.1126/sciadv.abl9404

The PDF file includes:

Figs. S1 to S9

Other Supplementary Material for this manuscript includes the following:

Data files S1 to S6

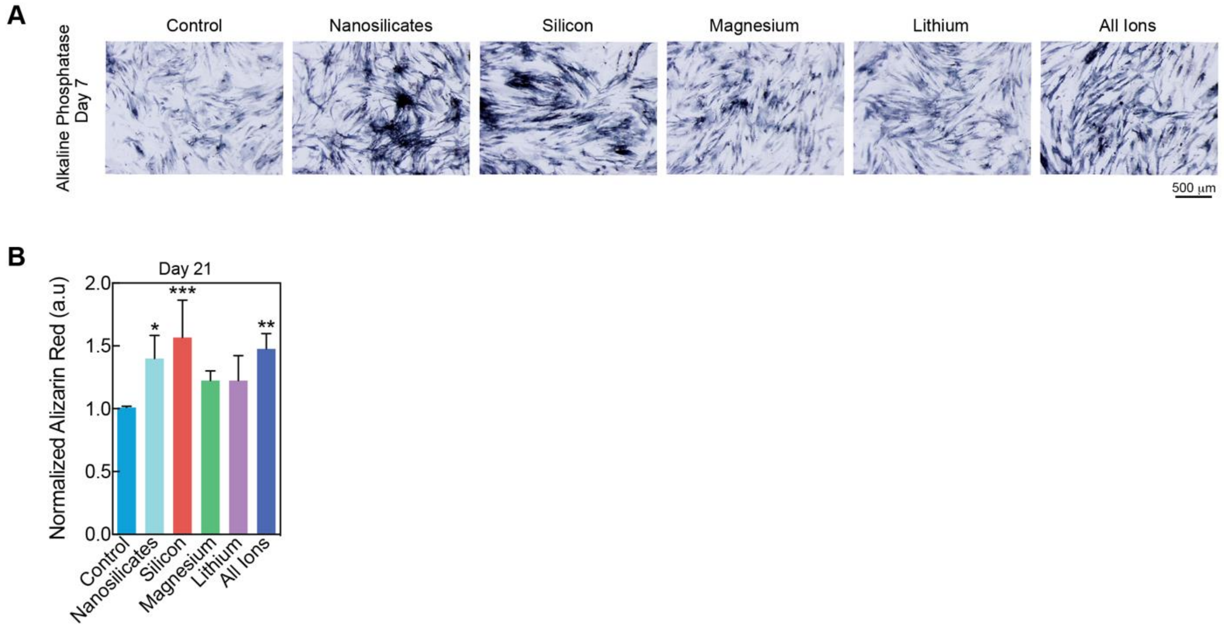

Fig S1: (**A**) Qualitative assessment of production of alkaline phosphatase (ALP) (day 14) due to treatment with nanosilicates and its ionic dissolution products. Blue precipitates represent intracellular ALP (stained using p-nitrophenyl phosphate, disodium salt). hMSCs cultured in osteoconductive media is used as a control.

(**B**) The effect nanosilicates and its ionic dissolution products was evaluated on production of mineralized ECM on day 21. The amount of calcium was quantified by dissolving the Alizarin Red S and quantifying via colorimetric assay.

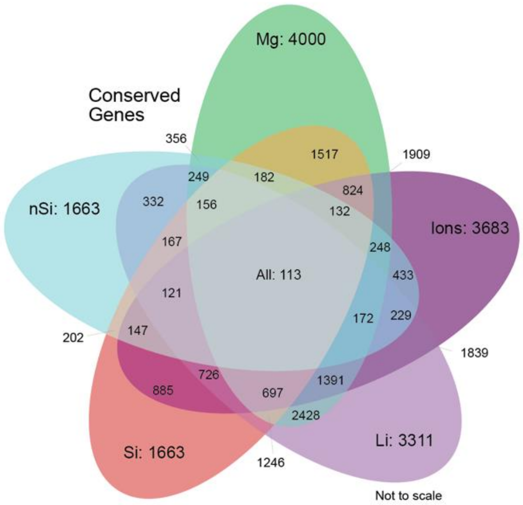

Fig. S2. Comparison of differentially expressed genes (DEGs) ($p < 0.05$) between treatments, and the amount of gene overlap between each treatment group. 113 DEGs are shared between all of the treatment groups.

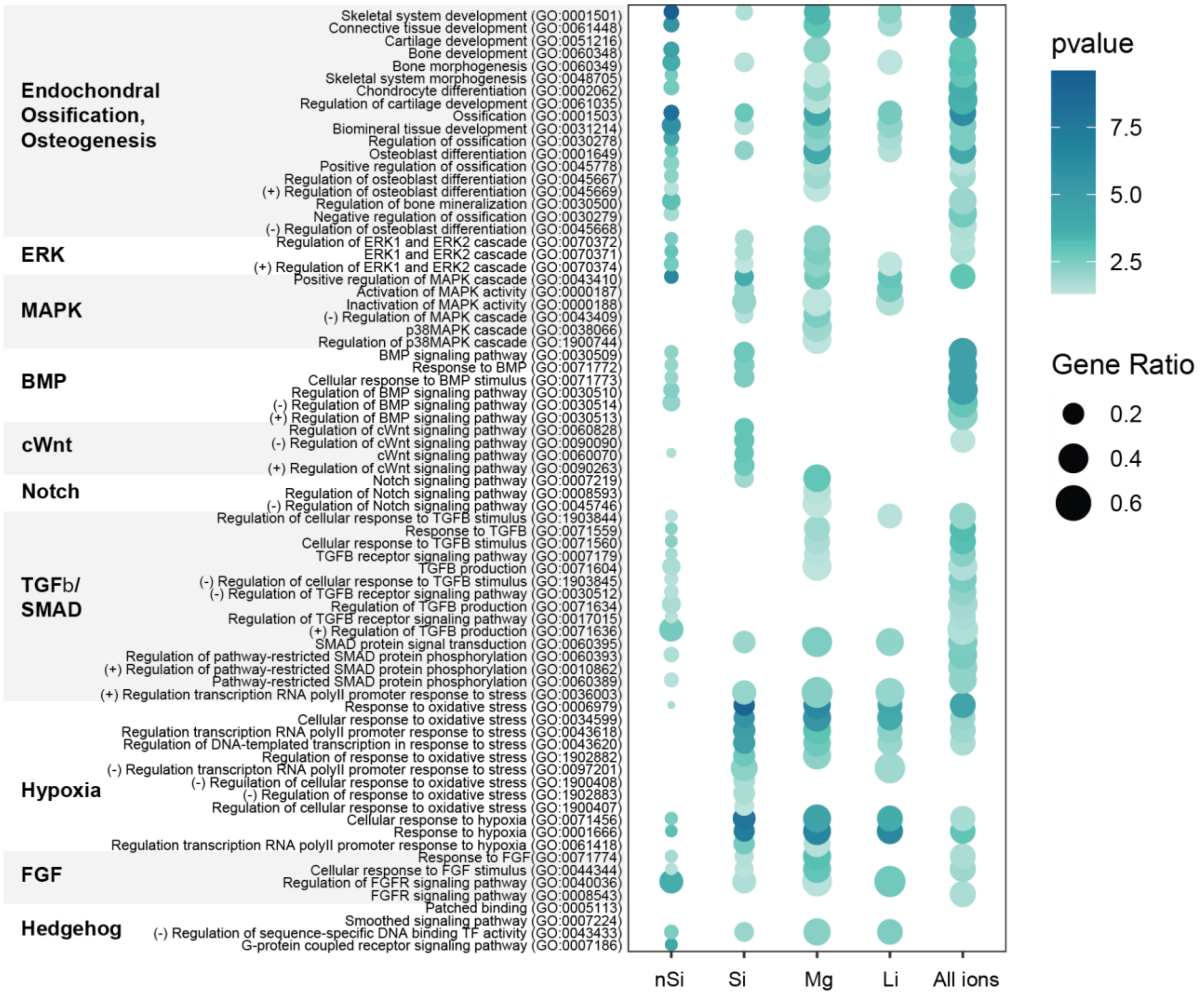

Fig. S3. Key GO terms associated with endochondral ossification and related pathways were selected. Perturbation of these key terms was evaluated for each nanoparticle treatment ($p < 0.05$). Circle size correlates to the gene ratio; i.e. the DEGs associated with a GO term divided by the total number of genes mapped to the GO term. Color intensity is associated with increasing -log(*p-adj*) (dark blue: greater significance, teal: less significance).

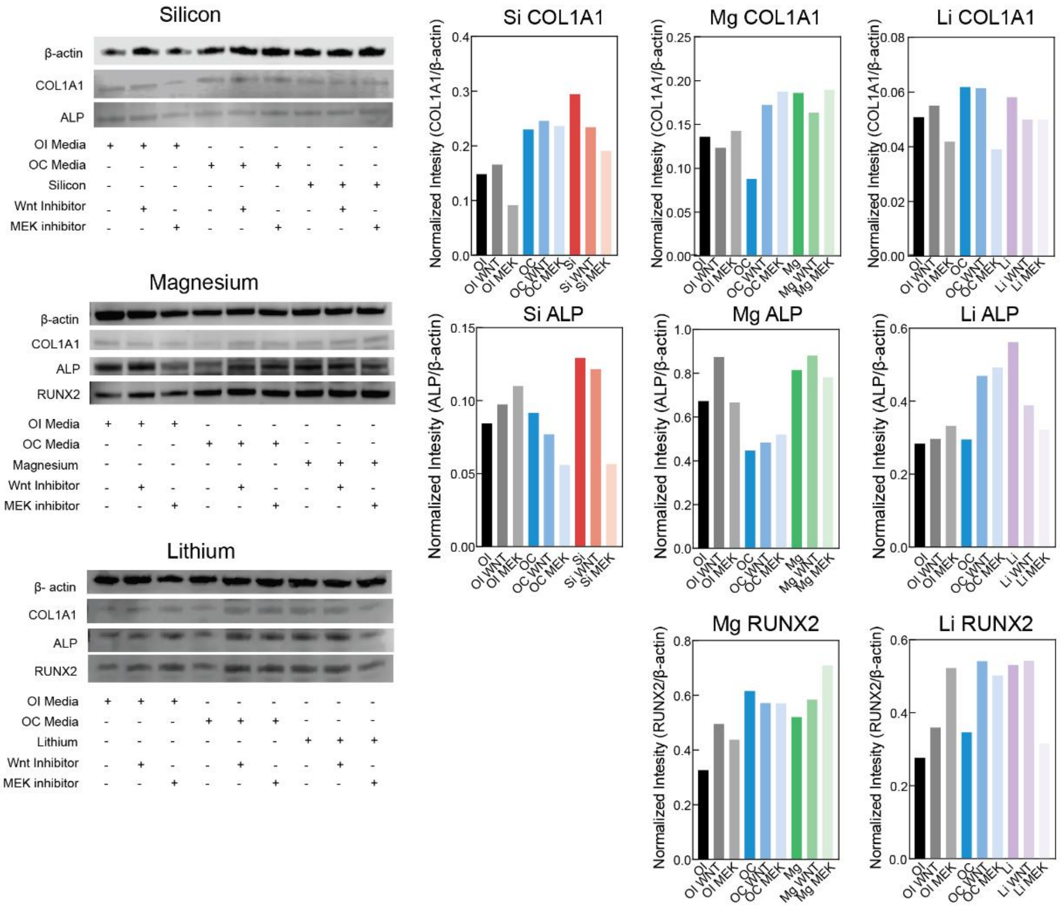

Fig. S4. Western blot of ALP protein expression of ion dissolution products compared to osteoinductive and osteoconductive controls treated with and without osteo-pathway inhibitors cWnt (10 µM cardamonin) and MEK (5 µM, PD185342) after 14 days. Quantification of protein expression from western blot normalized to β-actin expression.

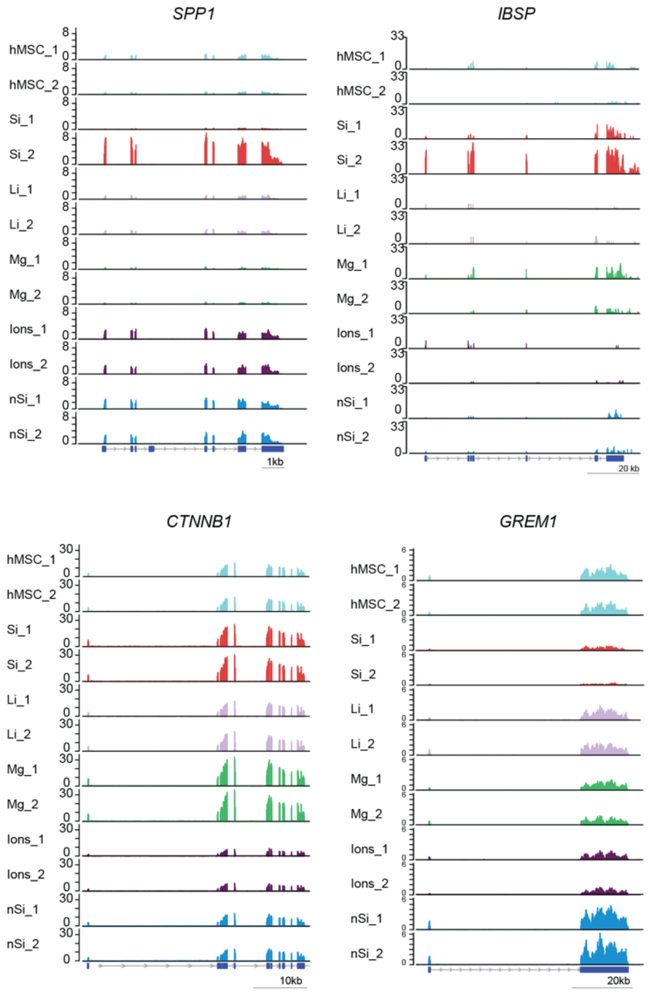

Fig. S5. Gene tracks of endochondral-related genes for each treatment group (nSi, Si, Mg, Li, and All Ions) at day 21 in hMSCs. Gene tracks of *SPP1* (osteopontin), an osteospecific glycoprotein, highlight an increase in expression for Si, nSi, and treatments with the combination of all ions. Gene tracks of a glycoprotein associated with mineralization, *IBSP* (bone-sialoprotein), indicate that Si treatment results in increased expression of this gene at day 21. Gene tracks of the hypertrophic chondrocyte marker *COL10A1* (collagen type 10 alpha 1 chain) showed an increased expression in samples treated with Li, Mg, and decreased expression in samples treated with the combination of individual ions and nSi. Gene tracks of the cWnt pathway component *CTNNB1* (β-catenin) showed an increased expression in samples treated with Si and Mg, and a decreased expression in samples treated with the combination of individual ions. Finally, the hypertrophic chondrocyte marker previously suggested to signify pre-osteoblastic behavior *GREM1* (gremlin 1) expression was increased in samples treated with nSi, and decreased in samples treated with Si, Mg, and the combination of all ions.

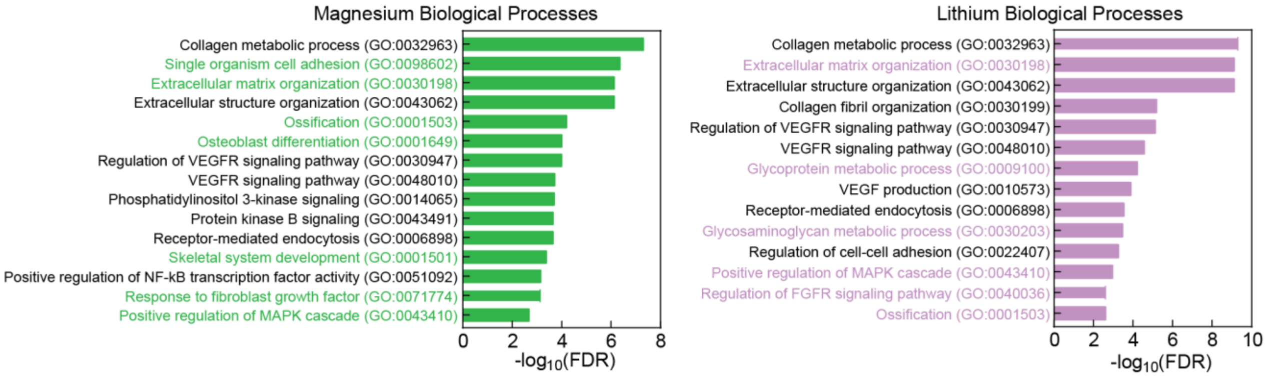

Fig. S6. Slimmed down graphs of significant ($p < 0.05$) GO terms in hMSCs treated with Mg or Li. These terms were identified through REVIGO and selected for association with endochondral ossification. Both Mg and Li regulate processes associated with FGF and VEGF signaling, which may be related to the observed matrix mineralization in these samples.

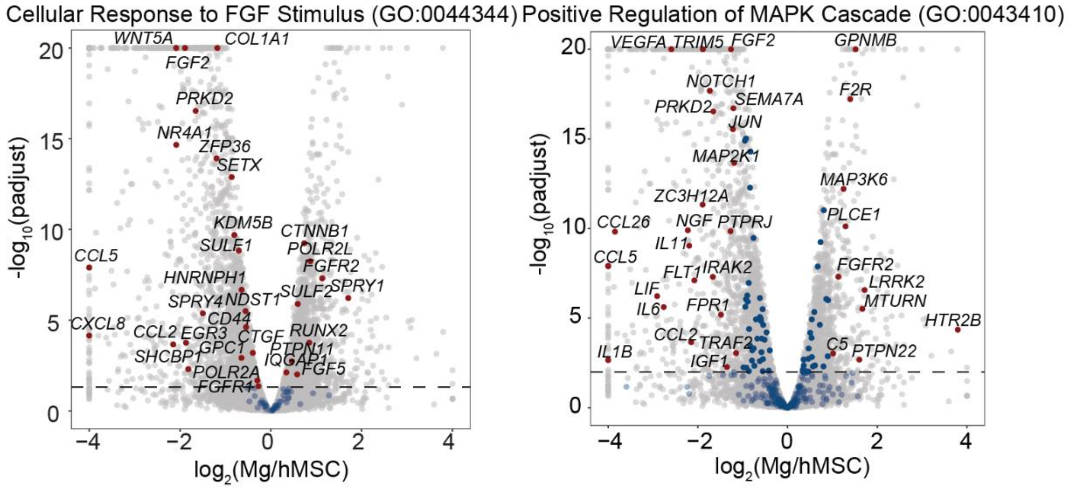

Fig. S7. Volcano plots of the GO term for positive regulation of MAPK Cascade (GO:0043410) and cellular response to FGF stimulus (GO:0044344). Both GO terms contain the FGF receptor 2 (FGFR2) DEG which has a positive fold change. This suggests that MAPK cascade may be positively regulated by a fibroblast growth factor (FGF) dependent mechanism. Further, within GO:0044344 the osteo-specific transcription factor RUNX2 is positively expressed, connecting FGF signaling to osteogenesis transcript expression.

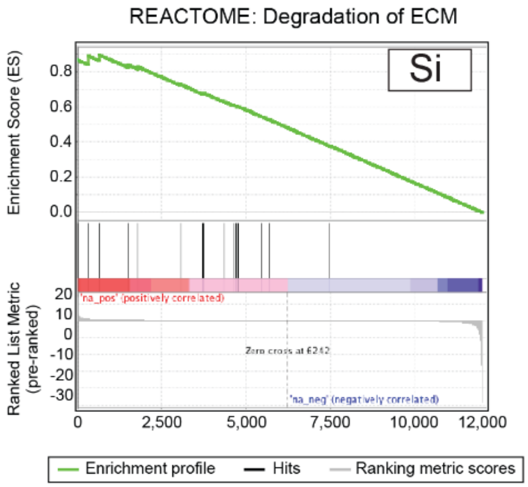

Fig. S8. The GSEA enrichment plot of silicon's effect on the *Reactome: Degradation of Extracellular Matrix* gene set, showing silicon results in a positive NES value of 1.85 and an adjusted *P*-value of 0.05. Core enriched genes in this gene set include positively and significantly correlated matrix metalloproteinases 11 and 15.

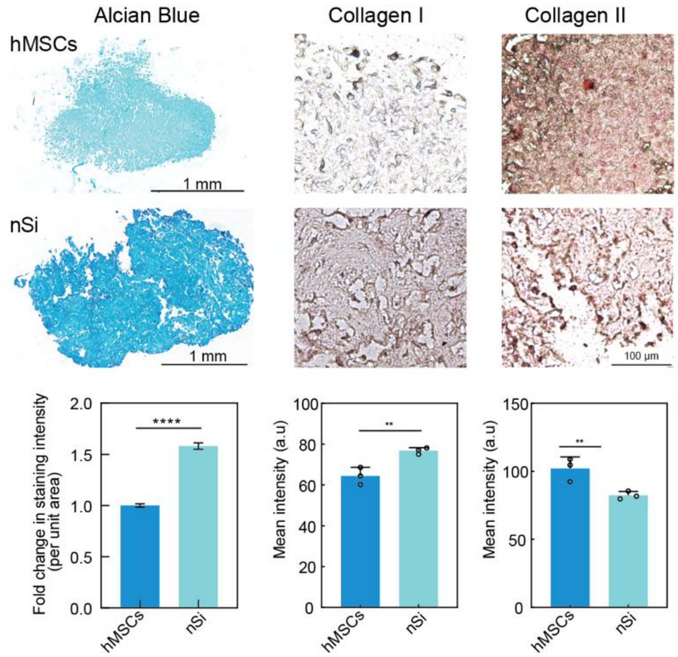

Fig. S9. hMSCs spheroids cultured in serum free media with and without nanosilicates treatment. After 30 days, the spheroids were sectioned and stained for Alcian blue, collagen type I and collagen type II. The images were normalized and quantified using imageJ. nSi treatment resulted in significantly (****$p < 0.0001$) increased GAG and Collagen I production compared to the negative control. A significant decrease in collagen II production was observed, supporting the ability of nanosilicates to support endochondral differentiation.
